# Highly efficient degradation of basic dyes using gold-coated nature-based supermagnetic iron oxide nanoparticles as eco-friendly nanocatalysts

**DOI:** 10.1007/s11356-024-32775-3

**Published:** 2024-03-09

**Authors:** Ghassan H. Matar, Muberra Andac

**Affiliations:** 1 Department of Chemistry, Ondokuz Mayis University, Samsun, Turkey; 2 Department of Nanoscience and Nanotechnology, Ondokuz Mayis University, Samsun, Turkey

**Keywords:** Green synthesis, Supermagnetic, Iron oxide nanoparticles, Gold-iron oxide bimetallic, Photocatalytic activity, Radical scavenging

## Abstract

Nowadays, organic dyes are prevalent components in wastewater discharges due to their extensive use in various industries, posing a significant threat to public health across different organisms. As a result, wastewater treatment has become an indispensable requirement. In this study, we synthesized supermagnetic iron oxide (Fe_3_O_4_ NPs) and gold-iron oxide bimetallic nanoparticles (Au@Fe_3_O_4_ BNPs) using an eco-friendly method that involved natural compounds extracted from brown Egyptian propolis. We employed UV-visible spectroscopy, FTIR, XRD, VSM, SEM, HRTEM, EDX, Zeta potential and XPS techniques to examine the optical characteristics, chemical structure, crystalline structure, magnetic properties, morphology, size, and chemical composition of these biosynthesized nanoparticles. Furthermore, these nanoparticles were used as nanocatalysts for the removal of cationic dyes. The photocatalytic results indicated high efficiency in the removal of methylene blue (MB), crystal violet (CV), and malachite green (MG) dyes from aqueous solutions using Fe_3_O_4_ NPs and Au@Fe_3_O_4_ BNPs. The removal rates of MB, CV, and MG were about 95.2% in 70 min, 99.4% in 50 min, and 96.2% in 60 min for Fe_3_O_4_ NPs, and 97.1% in 50 min, 99.1% in 30 min, and 98.1% in 50 min for Au@Fe_3_O_4_ BNPs, respectively. The study also assessed the potential anti-radical properties of the extract, Fe_3_O_4_ NPs, and Au@Fe_3_O_4_ BNPs using the DPPH assay, and the results demonstrated their antioxidant activity. Finally, these Fe_3_O_4_ NPs and Au@Fe_3_O_4_ BNPs have the potential to serve as efficient antioxidants and photocatalysts for removing basic dyes from water.

**Graphical abstract:**

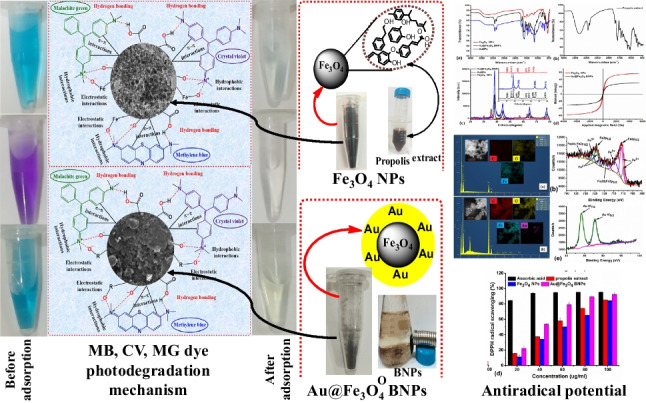

**Supplementary Information:**

The online version contains supplementary material available at 10.1007/s11356-024-32775-3.

## Introduction

Water resources, particularly groundwater, have become contaminated due to natural and artificial factors, as well as the release of various pollutants from different sources. Among the pollutants, dyes have emerged as a major concern, commonly carried by effluents from various industries, especially the textile industry (Kumar et al. 2022). Methylene blue (MB), crystal violet (CV), and malachite green (MG) are synthetic dyes widely employed in various industrial and laboratory applications (Oladoye et al. 2022). Despite their versatility and utility, these dyes have raised significant environmental concerns due to their potential to cause harm to ecosystems. MB and CV, when released into the environment through textile and industrial processes, can have adverse effects on water quality and aquatic life (Mani and Bharagava 2016). In particular, MG has been associated with environmental damage when used in aquaculture to treat fish diseases, as it can persist in aquatic environments and accumulate in fish tissues, posing risks to both aquatic organisms and human consumers (Kwan et al. 2020).

To minimize the risks of these diseases, it is essential to eliminate these pollutants from the water. Studies have revealed that untreated industrial effluents are one of the primary sources of these contaminants, which significantly affect the ecosystem and living organisms. Several technologies, time-consuming and non-destructive, are currently employed such as ion-exchange, adsorption, chemical precipitation, and membrane systems (Ahmed et al. 2022; Younas et al. 2021). Therefore, scientists are now developing environment-efficient technologies by using nanomaterials that demonstrate extensive potential for degrading hazardous organic dyes in water-based environments.

Nanotechnology as a highly concerned technology is increasingly used in environmental care (Shinde et al. 2023). In recent times, magnetic nanoparticles (MNPs) and bimetallic nanoparticles (BNPs) have gained significant attention due to their supermagnetic characteristics resulting from their small size (Idris and Roy 2023). These magnetic properties allow the potential use of these materials in eliminating and controlling environmental pollutants, as well as in the purification and prevention of their dispersion. Magnetite (Fe_3_O_4_) serves as an illustrative instance within this category and finds widespread use due to its robust magnetic attributes, expansive surface area, and limited toxicity, rendering it suitable for applications in both biomedicine and environmental remediation (Díez et al. 2022). These characteristics are contingent upon the methodology employed in the synthesis of MNPs, as it governs their dimensions, size distribution, shape, and consequently, their magnetic behavior. The characteristics of BNPs are influenced by various factors, including the type of reducing agents employed, the redox potentials of the metal ions, and van der Waals attractions among metal atoms (S. Ali et al. 2021). These factors determine whether BNPs will take on configurations such as core-shell, hetero-structure, multi-shell, cluster-in-cluster, or random alloy (Sharma et al. 2019). Additionally, the synthesis method used can impact the crystal structure and distribution of metal ions within the BNPs (F. Ali et al. 2023; N. Zhang et al. 2021). Consequently, the development and utilization of MNPs and BNPs, represent a promising avenue in the quest to address environmental challenges and advance various applications in environmental care (Cheng et al. 2023) and biomedicine (Allam et al. 2021).

Lately, there has been significant interest in the scientific community regarding green synthetic routes for producing nanoparticles (NPs). Many case studies focusing on green synthesis emphasize the use of methods and reagents that minimize the need for toxic reagents, solvents, or by-products during NPs preparation (Devi et al. 2023). The green approach, utilizing bio-active compounds and phytochemicals found in natural biomass, especially plant-based biomass like leaves, flowers, and flowers, has become the most commonly adopted method for preparing and stabilizing NPs (Laib et al. 2023; Rahman et al. 2022). This approach is straightforward, cost-effective, environmentally friendly, and safe for human use, making it highly advantageous for diverse NPs applications. Furthermore, being a one-step process, it also facilitates large-scale production.

Brown Egyptian propolis, derived from resinous substances collected by honeybees from various plant sources in Egypt, has garnered increasing attention for its diverse applications and numerous advantages. This type of propolis contains a complex mixture of bioactive compounds, including flavonoids, phenolic acids, and terpenes, which contribute to its potent antioxidant, antimicrobial, and anti-inflammatory properties (Matar and Andac 2023; Mohamed et al. 2022). These bioactive constituents have been harnessed for medicinal and therapeutic purposes, particularly in traditional and complementary medicine. However, the bio-fabrication of supermagnetic iron oxide nanoparticles (Fe_3_O_4_ NPs) and gold-iron oxide bimetallic nanoparticles (Au@Fe_3_O_4_ BNPs) using propolis extract has not been previously documented.

The current study was designed to address this gap by synthesizing Fe_3_O_4_ NPs and Au@Fe_3_O_4_ BNPs using Egyptian propolis extract. The synthesized Fe_3_O_4_ NPs and Au@Fe_3_O_4_ BNPs were then thoroughly characterized to confirm their particle size, morphology, crystallite size, particle composition, functional groups on the particle surface, and magnetic properties. Also, their photocatalytic potential for degrading basic dyes such as MB, CV, and MG dye as well as antiradical potential were tested. The successful utilization of propolis extract in synthesizing NPs could pave the way for further advancements and discoveries, making these NPs highly versatile for diverse applications in different scientific domains.

## Materials and methods

### Materials

The propolis samples were collected in the delta region, Egypt. Iron (III) chloride hexahydrate (FeCl_3_·6H_2_O), Iron (II) chloride tetrahydrate (FeCl_2_·4H_2_O), ammonium hydroxide solution (NH_4_OH, 28%), Tetrachloroauric (III) acid (HAuCl_4_·3H_2_O), and DPPH (1,1-Diphenyl-2-picryl-hydrazyl) were purchased from Sigma-Aldrich, USA. MB, CV, and MG were purchased from INTERLAB Laboratory Products, Turkey. Deionized water was used in all experiments.

### Green synthesis of AuNPs, Fe_3_O_4_ NPs, and Au@Fe_3_O_4_ BNPs

Green synthesis of monometallic and BNPs using propolis extract involves the use of natural compounds present in propolis as reducing and stabilizing agents.

#### Fe_3_O_4_ NPs biosynthesis

Initially, a propolis extract was prepared using a 90% ethanol solvent at a 1:10 (w/v) ratio, according to our previous work (Matar and Andac 2023). Regarding the biosynthesis of supermagnetic Fe_3_O_4_ NPs, 5 mL of the extract was added to 50 mL of FeCl_3_·6H_2_O (0.2M) and FeCl_2_·4H_2_O (0.1M) solutions. The yellowish-colored solution turned black with the addition of extract. Subsequently, 5 mL of NH_4_OH (28%) was added dropwise, and the solution was vigorously stirred for 1 h. The precipitated Fe_3_O_4_ NPs were collected using a magnet, washed with deionized water several times, and then dried at 50 °C in an oven. Later, the powder Fe_3_O_4_ NPs was stored at room temperature for further use. The following experimental steps for the biosynthesis of Fe_3_O_4_ NPs using propolis extract are presented in Fig. S1 (see Supplementary Material).

#### AuNPs biosynthesis

For propolis extract-mediated biosynthesis of AuNPs, 1 mL of the extract was added to a solution of HAuCl_4_·xH_2_O (1 mM, 50 mL), and the mixture was stirred and heated at 45 °C. The reducing agents present in the extract convert the gold ions into AuNPs as evidenced by the change in color of the solution to color dark purple. The synthesized AuNPs were washed three times with deionized water and then centrifuged at 8000 rpm for 15 min to remove unreacted biomolecules. Finally, the optimum time for green synthesis of AuNPs was recorded at 90 min. After that, the AuNPs were dried and stored at room temperature for further use.

#### Synthesis of Au@Fe_3_O_4_ BNPs

**Fig. 1:**
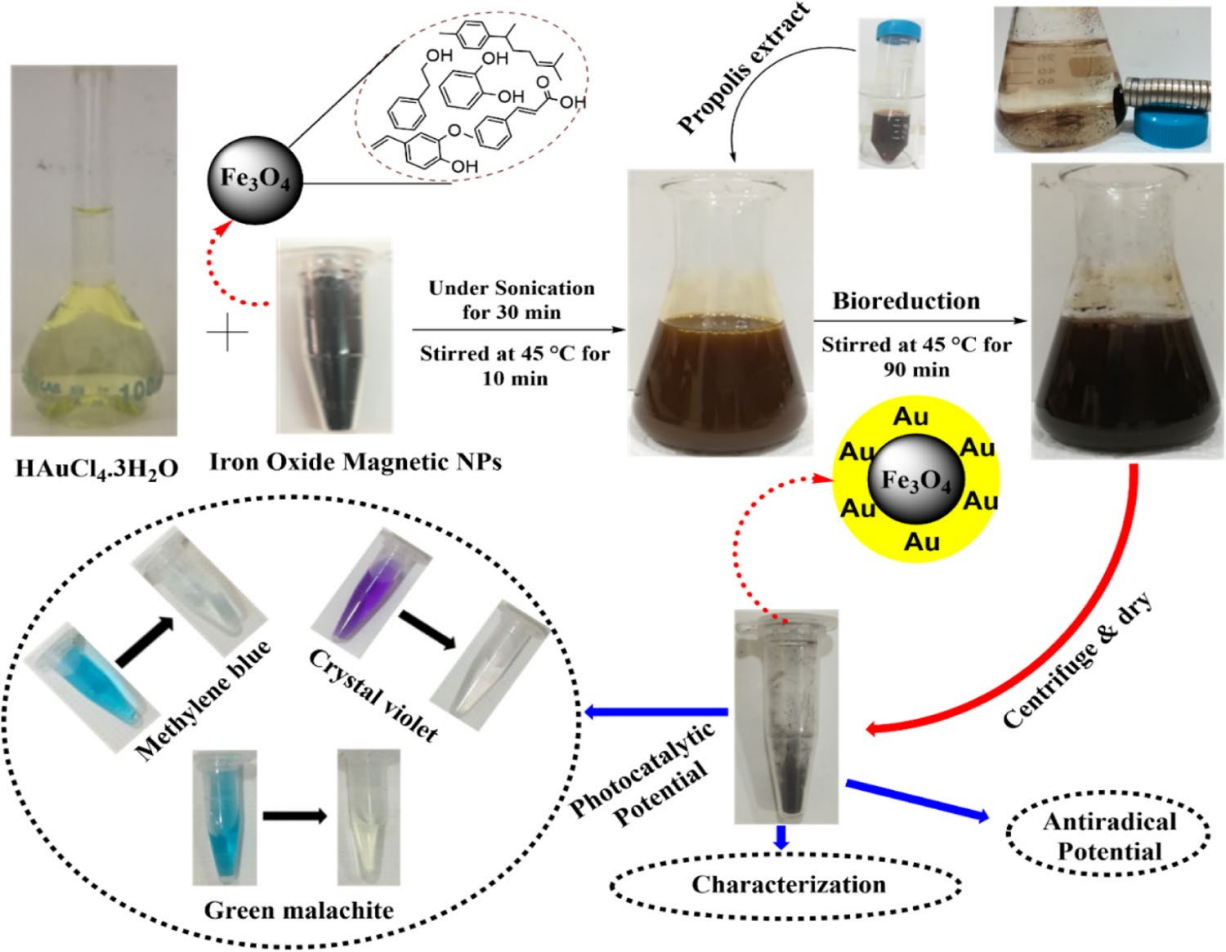
Schematic representation of the green synthesis of Au@Fe_3_O_4_ BNPs using propolis extract

Fe_3_O_4_ NPs synthesized as described in the previous step was used in the synthesis of Au@Fe_3_O_4_ BNPs. To achieve this, 0.1 g of the prepared Fe_3_O_4_ NPs was mixed with a 100 mL aqueous solution of 1 mM HAuCl_4_·3H_2_O under sonication for 30 min. The resulting brown-colored dispersion of Fe_3_O_4_ NPs in HAuCl_4_·3H_2_O solution was vigorously stirred at 45 °C for 10 min. 2 mL of propolis extract was then added drop by drop to the mixture, reducing the gold ions into AuNPs. The reaction was allowed to stir at 45 °C for 90 min. The brownish color solution changed to reddish-brown, indicating the formation and coating of AuNPs on the surface of the Fe_3_O_4_ NPs. After the synthesized Au@Fe_3_O_4_ BNPs were collected by a magnet, they were washed three times with deionized water and subsequently dried for further use. The following experimental steps for the biosynthesis of Au@Fe_3_O_4_ BNPs using propolis extract are shown in Fig. 1.

### Characterizations

The synthesis of Fe_3_O_4_ NPs, AuNPs, and Au@Fe_3_O_4_ BNPs, as well as their photocatalytic activities, were monitored by UV-visible spectroscopy (Thermo Scientific™, USA) at a wavelength ranging from 200-800 nm. FTIR spectroscopy (Perkin-Elmer Spectrum Two FT-IR) was used to determine functional groups on the surface of the Fe_3_O_4_ NPs, AuNPs and Au@Fe_3_O_4_ BNPs at wavelengths of 4000 – 400 cm^-1^. FE-SEM images (JEOL-JSM-7001F, Japan) and HR-TEM images (FEI TALOS F200S TEM 200 kV, USA) were deployed to examine the morphology and size of NPs. The elemental composition of surveyed samples was measured with EDX spectra (Oxford X-Max 80 mm^2^, UK). The crystalline structure of the Fe_3_O_4_ NPs and Au@Fe_3_O_4_ BNPs powder sample was measured using XRD (Rigaku Smart Lab X-ray diffractometer with monochromatic Cu Kα radiation, Japan) at 2θ ranging from 10° to 80°. The zeta potential of the Fe_3_O_4_ NPs and Au@Fe_3_O_4_ NPs was carried out by the zeta sizer instrument (Malvern Zetasizer equipped with MPT-2 Titrator, Nano ZS). The Fe_3_O_4_ NPs and Au@Fe_3_O_4_ BNPs powder sample were analyzed using a vibrating sample magnetometer (VSM) with the model Dexing Magnet VSM 550. The magnetization curve was recorded at room temperature. The chemical state of Fe_3_O_4_ NPs and Au@Fe_3_O_4_ BNPs was investigated using X-ray photoelectron spectroscopy (XPS, Thermo Scientific K-Alpha, USA).

### Photocatalytic degradation performance of basic dyes

The photocatalytic activities of MB, CV, and MG dyes on Fe_3_O_4_ NPs and Au@Fe_3_O_4_ BNPs were studied under direct light exposure. Typically, 10 mg of Fe_3_O_4_ NPs and Au@Fe_3_O_4_ BNPs were added to each of the dye solutions (10 mL, 10 ppm) without the addition of any catalyst. Subsequently, the reaction system was irradiated under visible light (a FCL 22W Daylight fluorescent lamp). The mixture was sampled every 10 min and collected using magnets. A UV-Vis spectrophotometer was used to examine the absorbance of the supernatant obtained with the magnets. Meanwhile, we performed the sorption process of dyes by keeping the mixtures in the dark for the same duration as the removal efficiency time observed in photodegradation for each dye. The degradation efficiency (%) was determined based on the maximum degradation of MB, CV, and MG dye solutions at 665 nm, 580 nm, and 618 nm, respectively. The efficiency of dye degradation was evaluated by applying equation (1).

1 $$\textrm{Degradation}\ \textrm{efficiency}\ \left(\%\right)=\left(\frac{\textrm{Ao}-\textrm{A}}{\textrm{Ao}}\right)\times 100$$

Here, A_o_ and A are the absorbance of dye before and after photo-irradiation, respectively.

### Antioxidant activity

The antioxidant activities of propolis extract, Fe_3_O_4_ NPs, and Au@Fe_3_O_4_ BNPs were evaluated using the DPPH free radical scavenging assay. In this assay, 0.5 mL of the propolis extract, Fe_3_O_4_ NPs, and Au@Fe_3_O_4_ BNPs at concentrations ranging from 20 to 100 μg/mL were mixed with 1 mL of a 0.1 mM DPPH solution. These mixtures were then incubated in darkness for 30 min. After incubation, the absorbance of these mixtures was measured at a wavelength of 517 nm. As a standard, ascorbic acid was used at the same concentration range. The percentage of free radical inhibition was determined using the following formula (2):

2 $$\textrm{DPPH}\ \textrm{rcavenging}\ \textrm{ability}\ \left(\%\right)=\left(\frac{\textrm{Ac}-\textrm{As}}{\textrm{Ac}}\right)\times 100$$

Here, Ac and As are the absorbance of control and after incubation of sample, respectively.

## Results and Discussion

### UV-visible spectrum analysis

**Fig. 2.**
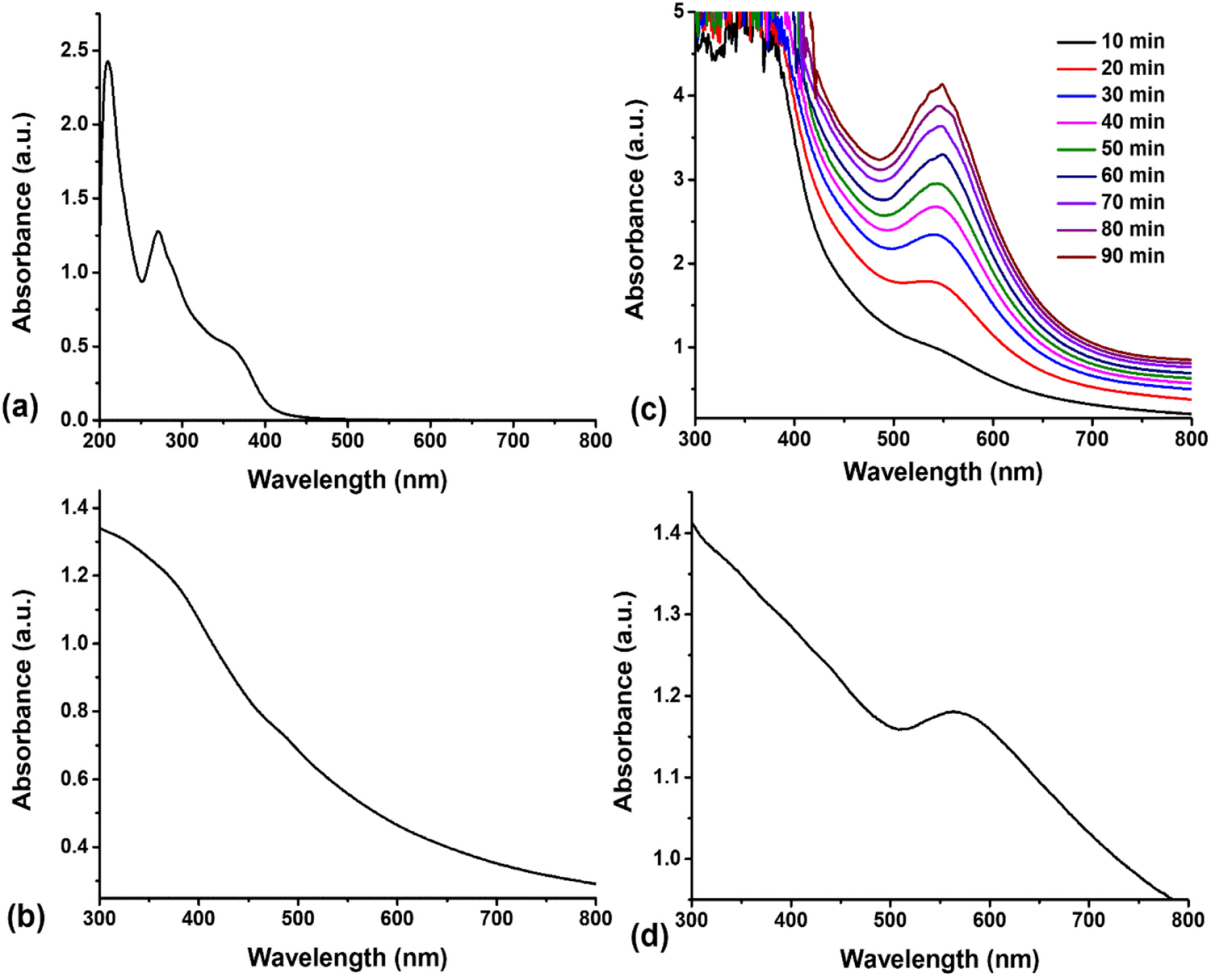
UV-visible spectrum of (**a**) the propolis extract, (**b**) Fe_3_O_4_ NPs, (**c**) the effect of reaction time on the green synthesis of AuNPs, and (**d**) Au@Fe_3_O_4_ BNPs

Figure 2 displays the UV-visible absorption spectrum of the propolis extract, Fe_3_O_4_ NPs, AuNPs, and Au@Fe_3_O_4_ BNPs. The UV-visible spectrum of the propolis extract exhibited a characteristic absorption peak at 270 nm (Fig. 2a), indicating the presence of phenolic and flavonoid compounds in the extract (Matar and Andac 2023). In contrast, the spectrum of Fe_3_O_4_ NPs (Fig. 2b) displayed no discernible absorption peak within the measured wavelength range, consistent with previous reports (Desai et al. 2023). To investigate the influence of incubation time on AuNPs synthesis, we monitored the UV-visible spectra at various time intervals (Fig. 2c). Remarkably, we observed a clear trend of increasing surface plasmon resonance (SPR) peak intensity with prolonged incubation time. The highest intensity of the SPR peak, recorded at a wavelength of 547 nm, was achieved at 90 min of incubation, which is consistent with previous reports (Abdelsattar et al. 2022). This result indicates that the synthesis of AuNPs was completed within 90 min, along with the associated processes of reduction and stabilization. In the spectrum of Au@Fe_3_O_4_ BNPs, the SPR peak was notably shifted to a longer wavelength (564 nm), confirming the successful synthesis of these BNPs, with the absorption peak consistent with the presence of AuNPs (Fig. 2d). This shift in the absorption of Au@Fe_3_O_4_ BNPs can be attributed to the attachment of AuNPs to Fe_3_O_4_ NPs. Furthermore, when we applied a magnetic field, both Fe_3_O_4_ NPs and Au@Fe_3_O_4_ BNPs displayed attraction towards the magnet (as shown in Fig. 1). This magnetic behavior, along with the shift in the characteristic peak from 547 to 564 nm, collectively confirmed the synthesis of Au@Fe_3_O_4_ BNPs. These findings align with prior research in the field, where a redshift in the SPR peak of Au@Fe_3_O_4_ BNPs is attributed to the dielectric properties of the Fe_3_O_4_ NPs (Desai et al. 2023).

### FTIR spectroscopy

**Fig. 3:**
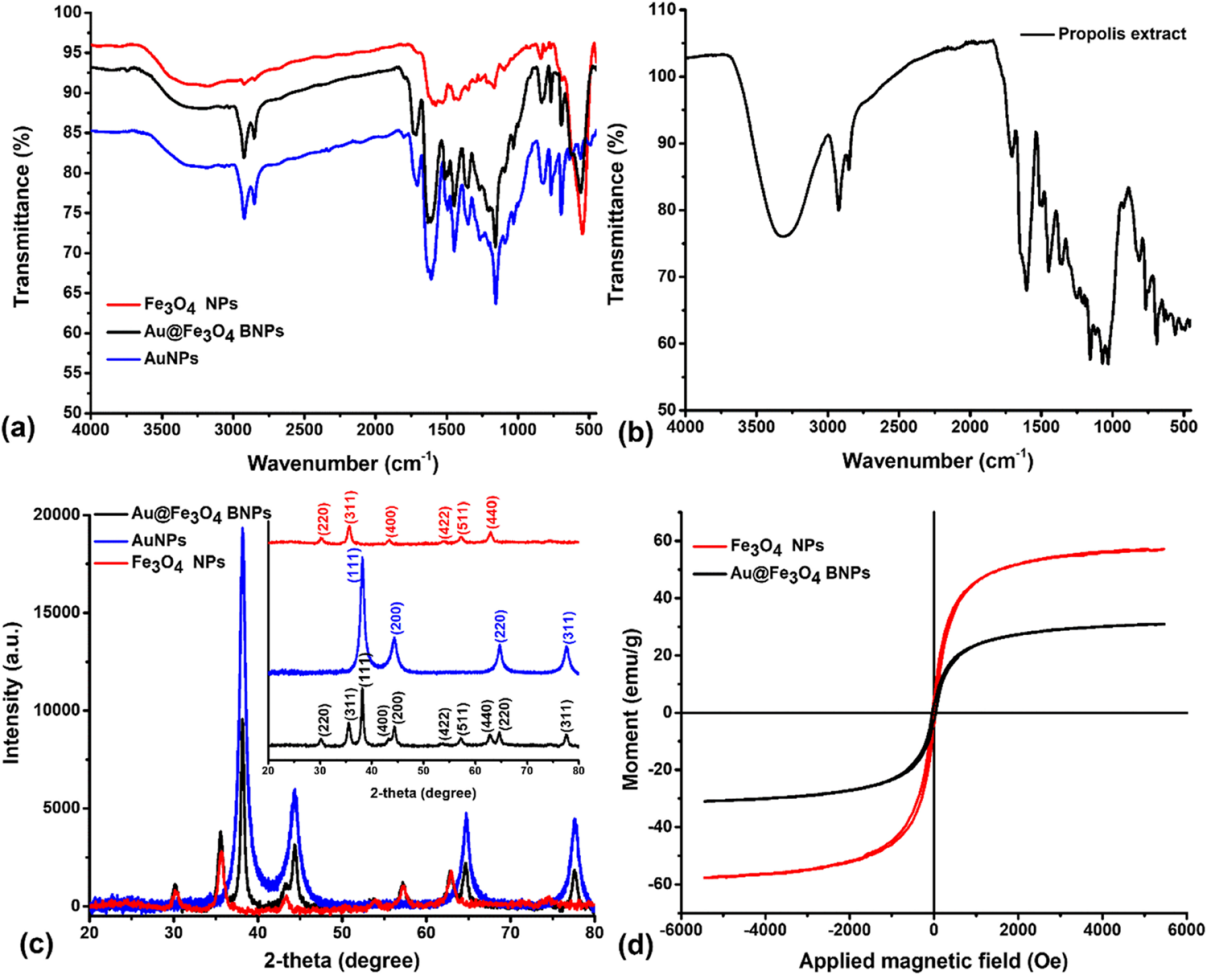
(**a**) FTIR spectra of purified Fe_3_O_4_ NPs, AuNPs and Au@Fe_3_O_4_ BNPs, (**b**) FTIR spectra of propolis extract, (**c**) XRD patterns of Fe_3_O_4_ NPs, AuNPs and Au@Fe_3_O_4_ BNPs, and (**d**) VSM analysis of Fe_3_O_4_ NPs and Au@Fe_3_O_4_ BNPs

The FTIR spectra of propolis extract, AuNPs, Fe_3_O_4_ NPs and Au@Fe_3_O_4_ BNPs are shown in Fig. 3a. The propolis extract exhibits a distinct peak at 3312 cm^-1^, attributed to the phenolic hydroxyl group. Meanwhile, other noticeable peaks at 2923, 2852, 1705, 1603, 1448, 1353, 1157, 1071 and 814, 767, 687 cm^-1^ (Fig. 3b) can be related to the C–H bond stretching, asymmetrical stretches of CH_2_, C=O stretching, C=C stretching, aromatic C–H, O–H bending, aromatic C–O bond stretching, stretching of C–O functional groups and C–H bending in the phenolic rings, respectively (Matar and Andac 2023). Along with the mentioned components, those can be derived from the secondary metabolites of the extract, in which polyphenols may facilitate the formation of NPs and prevent agglomeration. In the FTIR spectrum of Fe_3_O_4_ NPs, two distinct peaks at 3328 and 547 cm^-1^ are observed, associated with the characteristic vibrations of O–H and Fe–O, confirming the synthesis of Fe_3_O_4_ NPs (Desai et al. 2023). Additionally, the FTIR spectrum of Fe_3_O_4_ NPs displays narrow peaks at 1577, 1349, 1167, and 1097 cm^-1^, which can be attributed to the C=C stretching, O–H bending, aromatic C–O bond stretching, and stretching of C–O functional groups present in flavonoid compounds involved in biogenic synthesis. The FTIR spectrum of AuNPs exhibits characteristic peaks at 3215, 2922, 2851, 1706, 1608, 1447, and 1351 cm^-1^, corresponding to O–H stretching, C–H bond stretching, asymmetrical stretches of CH_2_, C=O stretching, C=C stretching, aromatic C–H, and O-H bending. Furthermore, the FTIR spectrum of AuNPs shows sharp peaks at 1156, 1030, and 821, 767, 696 cm^-1^, which can be attributed to the aromatic C–O bond stretching, stretching of C–O functional groups, and C–H bending in the phenolic rings present in flavonoid and phenolic compounds, which likely act as reducing or capping agents during the green synthesis. Similarly, in the FTIR spectrum of Au@Fe_3_O_4_ BNPs, the presence of a peak at 558 cm^-1^ indicates Fe–O bonding. The FTIR spectrum of Au@Fe_3_O_4_ BNPs exhibits peaks similar to the AuNPs spectra, with slight shifts in the distinct peaks to 3282, 2923, 2852, 1716, 1632, 1448, 1451, 1352, 1158, 1031, and 834, 768, 696 cm^-1^. These shifts are attributed to the biomolecules and functional groups responsible for attaching AuNPs to Fe_3_O_4_ NPs, thereby reducing and stabilizing them.

### XRD studies

The XRD pattern was utilized to examine the crystalline nature and size of the AuNPs, Fe_3_O_4_ NPs and Au@Fe_3_O_4_ BNPs. In Fig. 3c, the XRD pattern reveals strong and intense peaks with no additional impurity crystalline phases, indicating the high crystallinity of these NPs. For AuNPs, Bragg's reflections were observed at 2θ angles of 38.14°, 44.41°, 64.67°, and 77.43°, corresponding to the (111), (200), (220), and (311) crystal planes, respectively. This pattern is consistent with PDF card no. 00-004-0784, confirming the presence of AuNPs. In the XRD pattern of Fe_3_O_4_ NPs, Bragg's reflections at 2θ angles of 30.41°, 35.49°, 43.41°, 54.01°, 57.18°, and 62.88° corresponded to the (220), (311), (400), (422), (511), and (440) crystal planes, respectively. This pattern is consistent with PDF card no. 01-080-6403, confirming the presence of Fe_3_O_4_ NPs. Upon analyzing the XRD pattern of Au@Fe_3_O_4_ BNPs, reflections were found at 2θ angles of 30.16°, 35.57°, 38.15°, 43.24°, 44.37°, 53.77°, 57.22°, 62.78°, 64.58°, and 77.42°, corresponding to Fe (220), Fe (220), Au (111), Fe (400), Au (200), Fe (422), Fe (511), Fe (440), Au (220), and Au (311) crystal planes, respectively. The XRD pattern of Au@Fe_3_O_4_ BNPs was similar to both patterns of AuNPs and Fe_3_O_4_ NPs, indicating the formation of Au@Fe_3_O_4_ BNPs.

Furthermore, the average crystallite size of the NPs was estimated using the Debye-Scherrer (D-S) equation, Williamson-Hall (W-H) method, and Halder-Wagner (H-W) method as described in Equations (3-5) (Nath et al. 2020; Radoń et al. 2023):

3 $$D=\frac{K\lambda}{\beta\ cos\theta}$$

4 $$\beta Cos\theta =\frac{K\lambda}{D_{XRD}}+4\varepsilon Sin\theta$$

5 $${\left(\frac{FWHM}{tan\theta}\right)}^2=\frac{K\lambda}{D_{XRD}}\ \frac{FWHM}{tan\theta sin\theta}+{\left(\frac{\varepsilon }{2}\right)}^2$$

where D is the crystallite size (nm), K is the Scherrer constant (0.94 for spherical crystallites), λ is the X-ray wavelength (0.154 nm), β (FWHM) is the full width of half-maximum signal, and θ is the Bragg’s angle. Accordingly, the average crystallite size of the AuNPs, Fe_3_O_4_ NPs, and Au@Fe_3_O_4_ BNPs calculated to be 8.59, 11.66, and 14.28 nm, respectively (Table S1).

Also, the average crystallite size of the NPs was determined using the W-H method by plotting βcosθ against 4sinθ and fitting a straight line to the data. In Fig. S2a, b, and e, the slope of the line represents the strain (ɛ), and the y-intercept provides the crystallite size. The average crystallite size of the AuNPs, Fe_3_O_4_ NPs, and Au@Fe_3_O_4_ BNPs, calculated from the W-H plot, was 6.8, 12.9, and 10.7 nm, respectively (Table S2).

Similarly, the H-W method was used to determine the average crystallite size of the NPs by plotting ${\left(\frac{\textrm{FWHM}}{\textrm{tan}\uptheta}\right)}^2$ against $\frac{\textrm{FWHM}}{\textrm{tan}\uptheta \textrm{sin}\uptheta}$ (Fig. S2c, d, and f). The average crystallite size of the AuNPs, Fe_3_O_4_ NPs, and Au@Fe_3_O_4_ BNPs calculated from the H-W plot, was determined to be 6.7, 12.4, and 11.2 nm, respectively (Table S3). It’s noteworthy that the crystallite sizes obtained from both the W-H method and the H-W method are quite similar and also close to that of the S-D equation.

### Magnetic properties

The magnetic characteristics of biosynthesized Fe_3_O_4_ NPs and Au@Fe_3_O_4_ BNPs were assessed using a vibrating sample magnetometer (VSM). In Fig. 3d, it is demonstrated that the Au@Fe_3_O_4_ BNPs prepared in this study possess magnetic properties primarily attributed to the presence of Fe_3_O_4_ NPs. To verify their magnetic characteristics, we conducted measurements of hysteresis loops at a temperature of 25 °C with an applied magnetic field ranging from -6000 to +6000 Oe. The saturation magnetization (Ms), coercivity (Hc), and saturation remanence (Mr) parameters of Fe_3_O_4_ NPs and Au@Fe_3_O_4_ BNPs are listed in Table S4. The saturation magnetization (Ms) values for Fe_3_O_4_ NPs and Au@Fe_3_O_4_ BNPs were found to be 57.22 and 30.99 emu/g, respectively, confirming the supermagnetic nature of both Fe_3_O_4_ NPs and Au@Fe_3_O_4_ BNPs. The slight decrease in the Ms of Au@Fe_3_O_4_ BNPs can be attributed to the presence of Au on the outer surface of the Fe_3_O_4_ NPs. This outcome highlights the outstanding magnetic properties of these NPs in comparison to other magnetic NPs (Desai et al. 2023). Due to their impressive magnetic response, these biosynthesized Fe_3_O_4_ NPs and Au@Fe_3_O_4_ BNPs hold promise for diverse biomedical and environmental applications. In terms of environmental applications, the magnetic properties of the NPs contribute to an increased surface area available for adsorption. This phenomenon is crucial for enhancing the adsorption capacity of the NPs, making them more effective than their non-magnetic counterparts in removing dye molecules from aqueous solutions. Additionally, the magnetic properties of the NPs play a crucial role in their performance in antioxidant applications. The ability to guide and target these NPs using a magnetic field allows for the precise delivery of antioxidants to specific sites within biological systems, thereby preserving their stability and biocompatibility. This targeted approach enhances the therapeutic potential of the NPs in mitigating oxidative stress and related disorders (Moskvin et al. 2021).

### FE-SEM, HRTEM with SAED analysis and Zeta potential

**Fig. 4:**
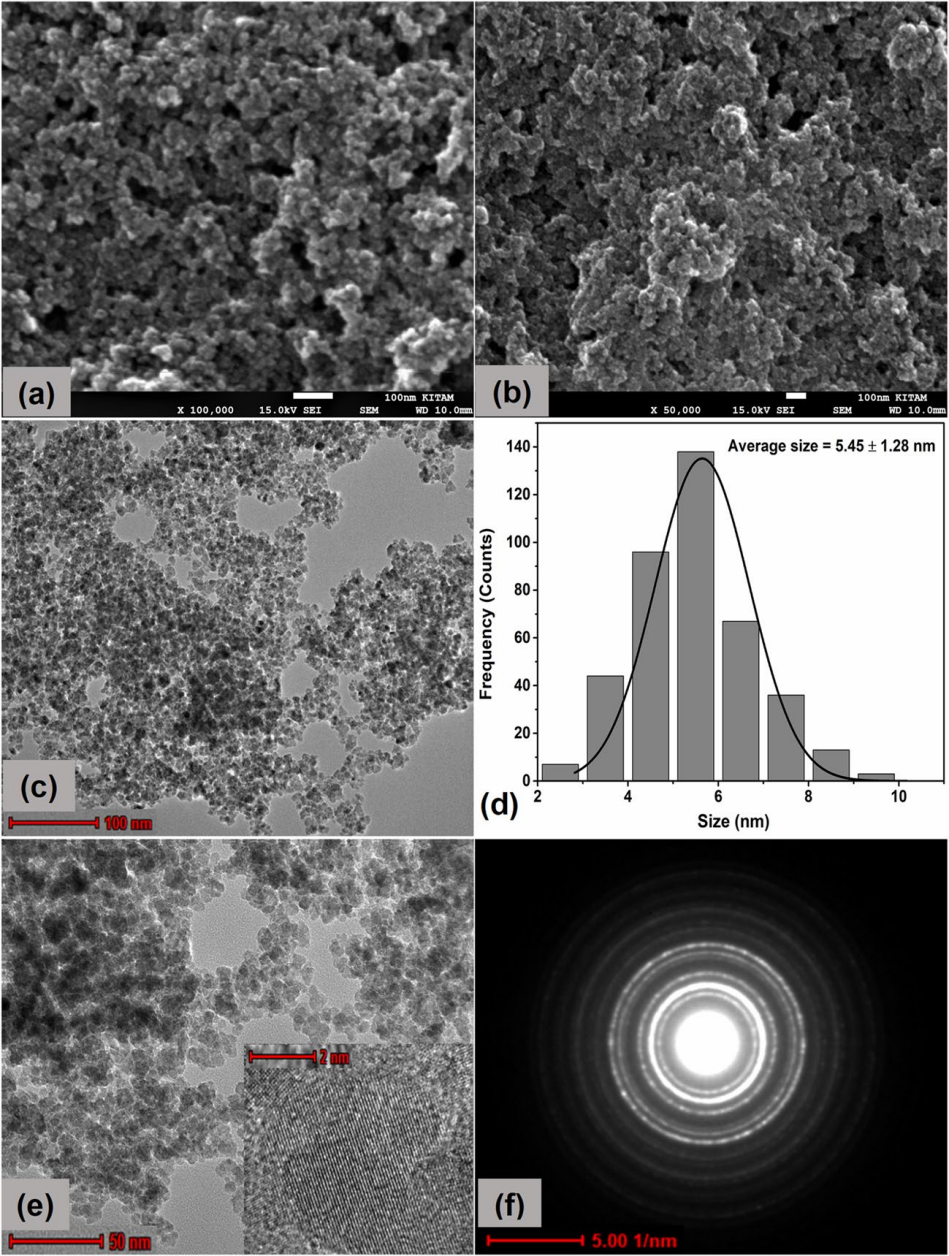
FESEM images at (**a**) 100 nm and at (**b**) 50 nm scale, TEM micrographs at (**c**) 100 nm scale, (**d**) particle size distribution, (**e**) HR-TEM image at 50 nm and showing fringe spacing at 2 nm scale, and (**f**) SAED pattern of biosynthesized Fe_3_O_4_ NPs

**Fig. 5:**
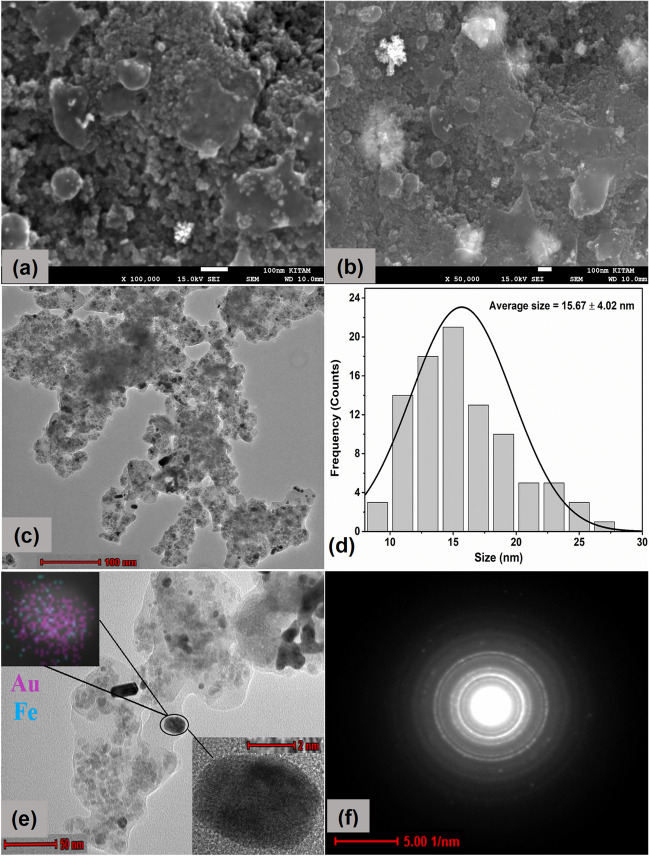
FESEM images at (**a**) 100 nm and at (**b**) 50 nm scale, TEM micrographs at (**c**) 100 nm scale, (**d**) particle size distribution, (**e**) HR-TEM image at 50 nm and showing fringe spacing at 2 nm scale, and (**f**) SAED pattern of biosynthesized Au@Fe_3_O_4_ BNPs

The morphology and particle size of Fe_3_O_4_ NPs and Au@Fe_3_O_4_ BNPs were examined using FE-SEM and TEM (Fig. 4 and Fig. 5). Fig. 4a-c and Fig. 5a-c display FE-SEM and TEM images of Fe_3_O_4_ NPs and Au@Fe_3_O_4_ BNPs at different magnifications. These images show that the synthesized Fe_3_O_4_ NPs had uniform and monodispersive spherical shapes of varying sizes. In contrast, the Au@Fe_3_O_4_ BNPs appeared aggregated with spherical, rectangular shapes, and slightly larger sizes. This uniform dispersion of particles may be attributed to the presence of bio-analytes on the particle surface, which acted as stabilizing agents. The analysis of the TEM micrograph showed that the biosynthesized Fe_3_O_4_ NPs and Au@Fe_3_O_4_ BNPs had an average size of 5.45 ± 1.28 nm and 15.67 ± 4.02 nm, respectively, as shown in the size distribution histogram (Fig. 4d and Fig. 5d). In Fig. 4e and Fig. 5e, HRTEM images at 2 nm show fine fringes within the Fe_3_O_4_ NPs and Au@Fe_3_O_4_ BNPs. Fig. 5e also depicts the homogeneous presence of Fe_3_O_4_ NPs and AuNPs in the HRTEM image of Au@Fe_3_O_4_ BNPs, as confirmed through EDX analysis.

**Fig. 6:**
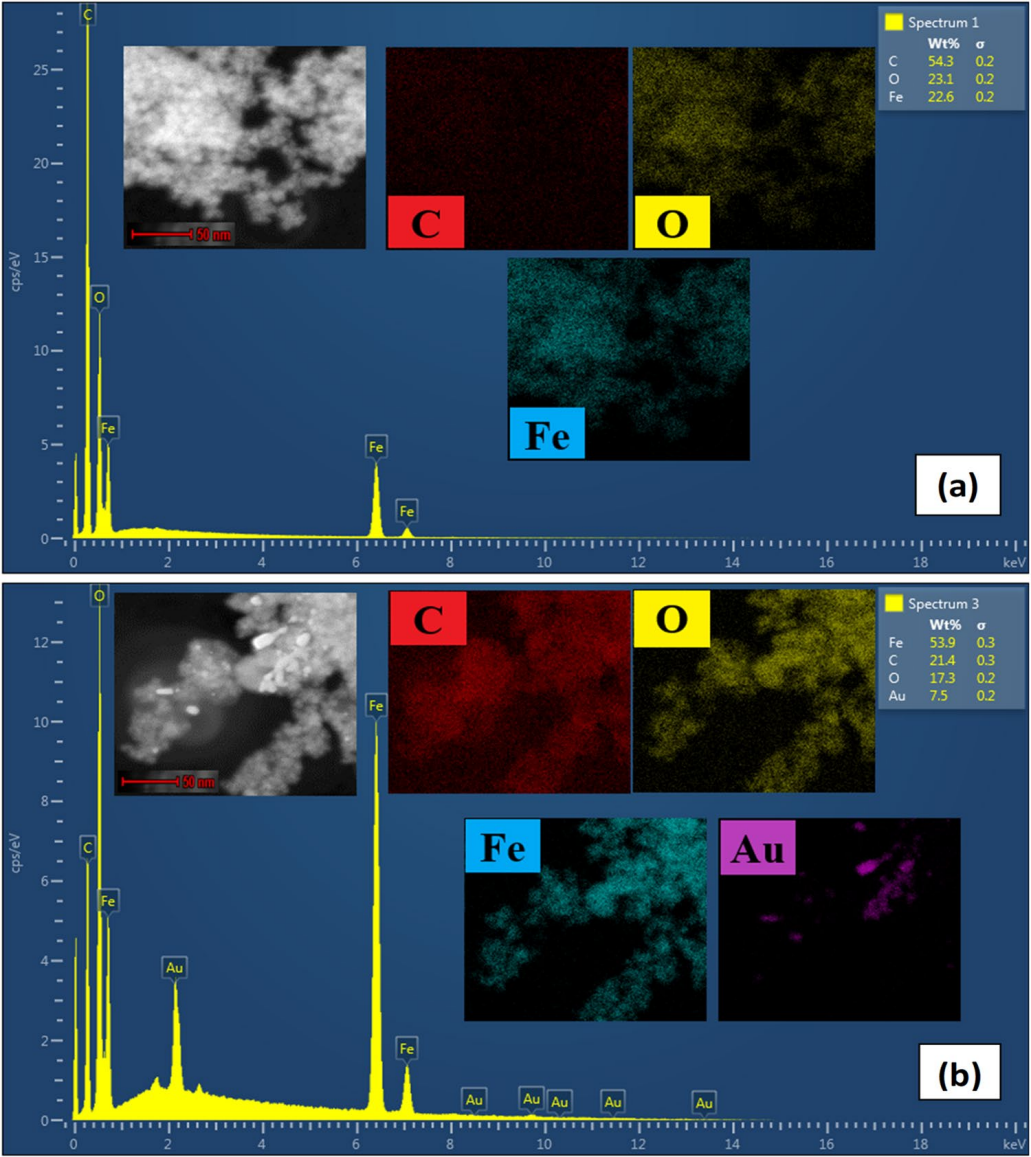
EDX spectrum and mapping images of (**a**) biosynthesized Fe_3_O_4_ NPs and (**b**) Au@Fe_3_O_4_ BNPs

Furthermore, SAED patterns were utilized to examine the crystalline nature of the synthesized Fe_3_O_4_ NPs and Au@Fe_3_O_4_ BNPs (Fig. 4f) and (Fig. 5f). The d-spacing of Fe_3_O_4_ NPs and Au@Fe_3_O_4_ BNPs was calculated through SAED patterns using Image J software (Table S5). The SAED patterns of the Fe_3_O_4_ NPs and Au@Fe_3_O_4_ BNPs indicated the presence of intense bright spots on the diffraction rings, confirming the polycrystalline nature of these NPs. The diffraction rings of Fe_3_O_4_ BNPs were found at interplanar distances of 0.293, 0.252, 0.205, 0.169, 0.160, and 0.146 nm, corresponding to the (220), (311), (400), (422), (511), and (440) planes of Fe_3_O_4_. Whereas the SAED pattern of Au@Fe_3_O_4_ BNPs in Fig. 6f clearly depicts diffraction spots corresponding to the (220), (311), (400), (422), (511), and (440) planes of Fe_3_O_4_, and the planes (111), (200), (220), and (311) were observed for Au. Thus, the measurements obtained from the SAED pattern very closely align with the measurements of XRD analysis.

The zeta potential of the Fe_3_O_4_ NPs and Au@Fe_3_O_4_ NPs showed a negative charge, -26.3 mV and -34.7 mV, respectively (Fig. S3). The notable negative zeta potential observed in Fe_3_O_4_ NPs and Au@Fe_3_O_4_ NPs is associated with their prolonged stability and colloidal structure. This negative charge plays a crucial role in preventing the agglomeration of nanoparticles in the solution by fostering repulsion among them. Since the Fe_3_O_4_ NPs and Au@Fe_3_O_4_ NPs have a negative charge, they are likely to attract and interact with cationic dyes. In this case, cationic dyes such as MB, CV, and MG dye, which carry a positive charge, would be attracted to the negatively charged surface of these nanoparticles. This electrostatic interaction between the negatively charged nanoparticles and the positively charged cationic dyes can lead to adsorption of the dyes onto the nanoparticle surface.

### EDX analysis

The elemental compositions of Fe_3_O_4_ NPs and Au@Fe_3_O_4_ BNPs were determined by EDX as shown in Fig. 6a, b. In Fig. 6a, the EDX spectrum of the Fe_3_O_4_ NPs indicates the predominant presence of carbon (C, 54.3 wt%), oxygen (O, 23.1 wt%), and iron (Fe, 22.6 wt%) within the NPs, confirming the successful synthesis of Fe_3_O_4_ NPs. In Fig. 6b, the EDX spectra reveal the presence of elemental carbon (C, 21.4 wt%), oxygen (O, 17.3 wt%), and iron (Fe, 53.9 wt%) along with gold (Au, 7.5 wt%) peaks, confirming the successful synthesis of Au@Fe_3_O_4_ BNPs.

Additionally, the EDX spectra of NPs indicate the presence of carbon and oxygen elements, attributed to extract role as the reducing agent in the biosynthesis process. In the mapping spectrum, the red, yellow, blue, and purple dots represent the distribution of C, O, Fe and Au elements within the NPs.

### XPS analysis

**Fig. 7:**
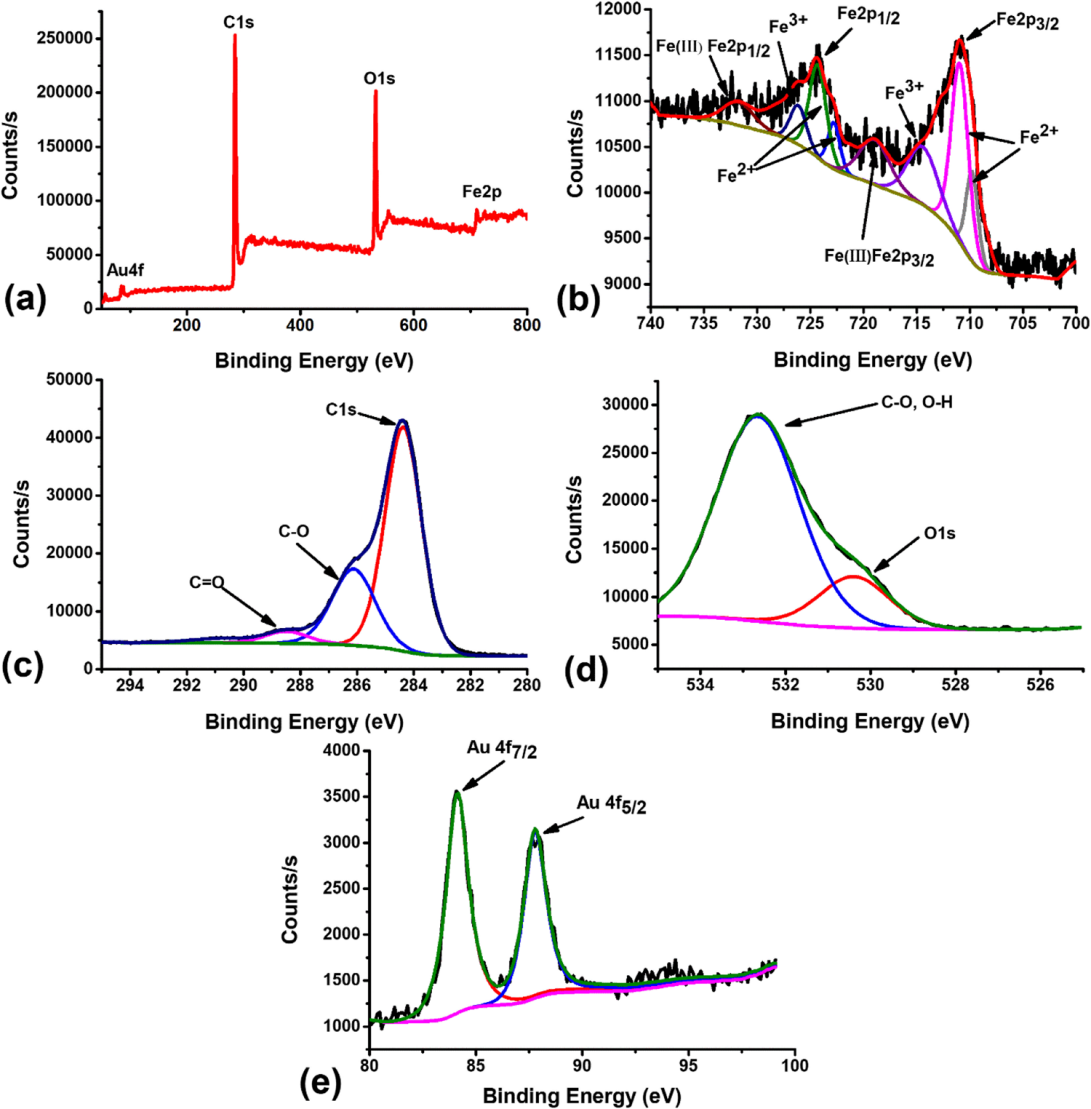
(**a**) XPS survey spectrum of Au@Fe_3_O_4_ BNPs, (**b**) Fe2p spectra, (**c**) C 1s spectra, (**d**) O 1s spectra, and Au 4f spectra

XPS was used to investigate the oxidation state and chemical composition of the Fe_3_O_4_ NPs and Au@Fe_3_O_4_ BNPs. Table S6 presents comprehensive data regarding the elemental composition obtained through XPS analysis. Fig. S4 and Fig. 7 show the XPS data for Fe_3_O_4_ NPs and Au@Fe_3_O_4_ BNPs. Fig. S4a presents the survey scan of the Fe_3_O_4_ NPs, confirming the presence of C, O, and Fe in these NPs, which is consistent with the results of the EDX analysis. The Fig. S4c and Fig. 7c show the C 1s spectra of Fe_3_O_4_ NPs. As observed, C 1s spectra can be deconvoluted into three peaks at 284.6, 286.2, and 288.5 eV, corresponding to (C-C, C-H), C-O, and C=O, respectively (Ravelo-Nieto et al. 2023). In Fig. S4d and Fig. 7d, the O 1s spectra can be deconvoluted into two peaks located at 530.1 and 531.0 eV, corresponding to the oxygen species in the Fe-O component of magnetite and oxygen in the C-O or O-H components (López et al. 2023). The presence of carbon and oxygen in the Fe_3_O_4_ NPs was attributed to the capping of propolis extract biomolecules. Furthermore, Fig. S4b and Fig. 7b display the narrow scan of the Fe 2p spectrum, confirming the presence of characteristic peaks such as Fe2p_3/2_ (710.7 eV) and Fe2p_1/2_ (724.4 eV) in the NPs. These peaks can be further subdivided into six peaks at 710.1, 711.2, 713.7, 723.2, 724.6, and 727.2 eV. The peaks at 710.1, 711.2, 723.2, and 724.6 eV indicate the presence of Fe-O bonds in the Fe^2+^ ion, while the peaks at 713.7 and 727.2 eV were associated with Fe-O bonds in the Fe^3+^ ion within the Fe_3_O_4_ (Rajan et al. 2020). In Fig. 7a, the XPS survey spectrum of Au@Fe_3_O_4_ BNPs confirms the presence of C, O, Fe, and Au, which is consistent with the results of the EDX analysis. Notably, the intensity of the C 1s peak increased while the O 1s peak decreased noticeably. This change was due to the presence of Au on the outer surface of the Fe_3_O_4_ NPs (Fe_3_O_4_ NPs coated with Au). Fig. 7e shows the Au 4f spectra of Au@Fe_3_O_4_ BNPs. As observed, the Au 4f spectra can be deconvoluted into two doublet peaks at 84.1 and 87.8 eV, corresponding to Au 4f_7/2_, and Au 4f_5/2_, respectively (Naraginti and Li 2017). Moreover, there was almost no difference between the positions of the peaks in the C 1s and Fe 2p spectra for both Fe_3_O_4_ NPs and Au@Fe_3_O_4_ BNPs. Meanwhile, the peak intensity of the O 1s for Au@Fe_3_O_4_ BNPs decreased significantly, indicating that more AuNPs were coated on the surface of Fe_3_O_4_ NPs, as well as highlighting the strong interaction between AuNPs and Fe_3_O_4_. Taken together, these results confirm the successful fabrication of Au@Fe_3_O_4_ BNPs.

### Evaluation of photocatalytic performance

In this section, we assessed the photocatalytic performance of Fe_3_O_4_ NPs and Au@Fe_3_O_4_ BNPs as nanocatalysts by investigating their ability to degrade the dyes MB, CV, and MG, which are considered as toxic pollutants. After adding Fe_3_O_4_ NPs and Au@Fe_3_O_4_ BNPs to the dye solution and exposing it to fluorescent lamp light, we recorded the photocatalytic results of MB, CV, and MG dyes at regular time intervals using Fe_3_O_4_ NPs and Au@Fe_3_O_4_ BNPs. Over time, we observed changes in the absorption spectra of MB, CV, and MG, with characteristic absorption wavelengths (λmax) at 665 nm, 580 nm, and 618 nm, respectively. The absorption peaks of these dyes decreased with time, further supported by the fading of the dye color, as shown in the insets of Fig. S5 and Fig. S6.

#### Degradation of MB, CV, and MG dyes using Fe_3_O_4_ NPs

**Fig. 8:**
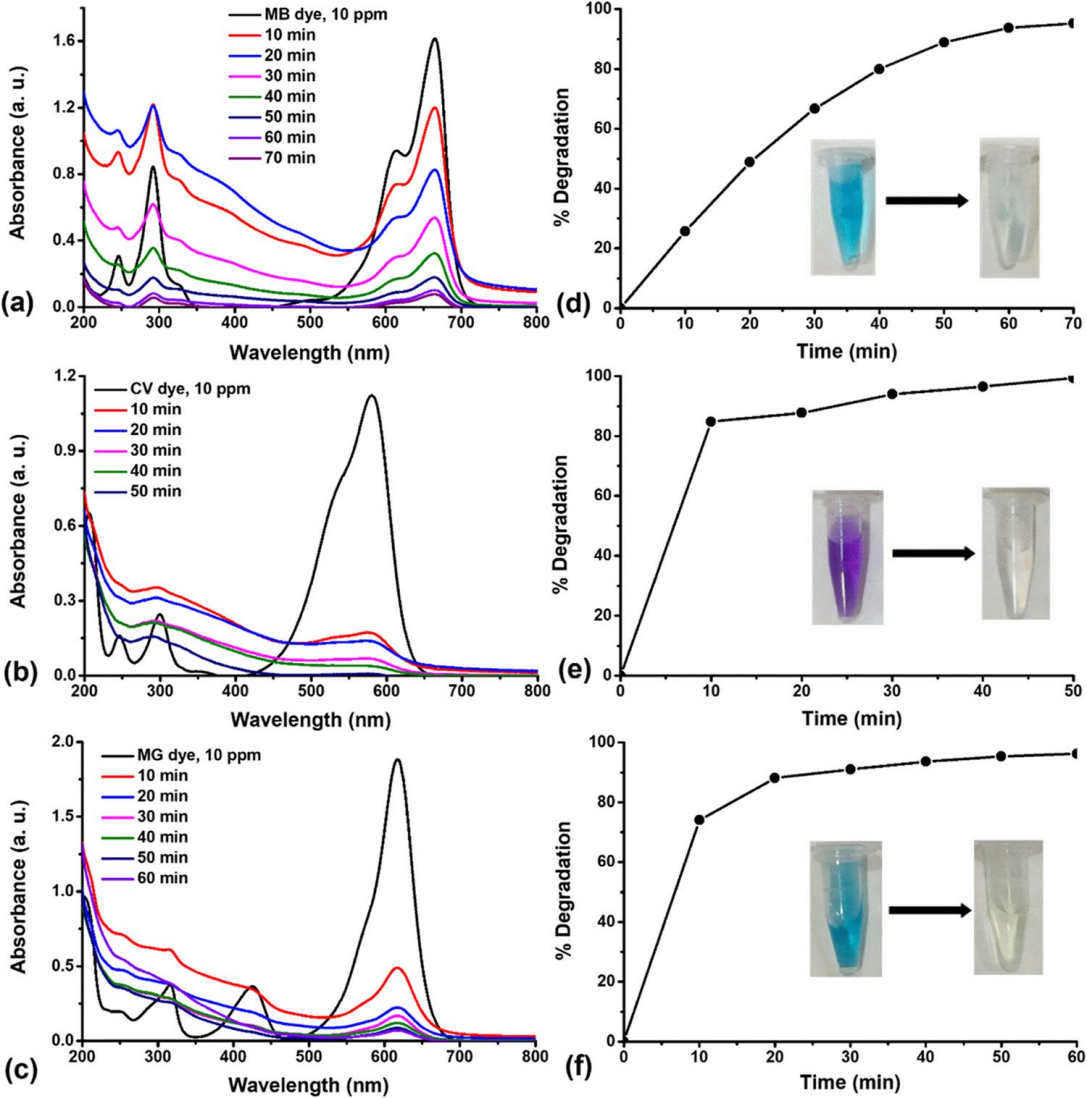
Absorption spectra of photocatalytic degradation of (**a**) MB, (**b**) CV, and (**c**) MG dye treated with Fe_3_O_4_ NPs at different time points and removal efficiency percentages of (**d**) MB, (**e**) CV, and (**f**) MG dye

The photocatalytic performance of biosynthesized Fe_3_O_4_ NPs to degrade MB, CV and MG dyes was examined under fluorescent lamp light at room temperature. Fig. 8 represents UV-visible absorption plots of photodegradation of MB, CV, and MG dye treated with Fe_3_O_4_ NPs at different time intervals and their degradation percentages. Increasing the contact time resulted in a higher percentage of dye removal for all dyes examined. The ideal contact times for removing MB, CV, and MG were determined to be 70 min, 50 min, and 60 min, respectively, leading to the highest levels of dye removal (Fig. 8a, b, c). As displayed in Fig. 8d, e, f, Fe_3_O_4_ NPs degraded the MB, CV, and MG dye at a concentration of 10 ppm with 95.2%, 99.4%, and 96.2% degradation efficiency, respectively. Meanwhile, the removal of MB, CV, and MG dyes using Fe_3_O_4_ NPs was performed in the dark for 70 min, 50 min, and 60 min, respectively, as shown in Fig. S7. Still, the removal percentage efficiency was relatively lesser compared to the degradation efficiency of MB, CV, and MG dye in the presence of light (Table S7). The removal efficiency time for the dark reaction was 20 h for MB, CV, and MG dyes, whereas the degradation efficiency time for the light reaction was 70 min, 50 min, and 60 min for MB, CV, and MG dyes, respectively. It is well known that MB, CV, and MG act as cationic dyes in the aqueous solution. The presence of certain forces, such as hydrogen bonding, electrostatic interactions and hydrophobic-hydrophobic interactions, plays a crucial role in regulating the attachment of MB, CV, and MG dye (Cheng et al. 2023). Synthesized Fe_3_O_4_ NPs were produced using propolis extract containing bioactive compounds such as flavonoids and phenolic compounds. Analysis of FTIR and XPS spectra revealed the presence of functional groups such as hydroxyl groups (–OH) and carbonyl groups (C=O) on the surface of Fe_3_O_4_ NPs. These functional groups can potentially interact with the functional groups of dyes (MB, CV, MG) through hydrogen bonding (Fig. 10a). Additionally, hydrophobic-hydrophobic interactions may form due to the hydrophobic surface of the Fe_3_O_4_ NPs, which facilitates increased interactions between the Fe_3_O_4_ NPs and (MB, CV, MG) dyes in an aqueous medium (Fig. 10a). Furthermore, the high surface area and high negative surface charge of the Fe_3_O_4_ NPs facilitate the physisorption of these dyes onto the Fe_3_O_4_ NPs, as well as increased electrostatic interaction between the Fe_3_O_4_ NPs and the dye (Fig. 10a).

#### Degradation of MB, CV, and MG dyes using Au@Fe_3_O_4_ BNPs

**Fig. 9:**
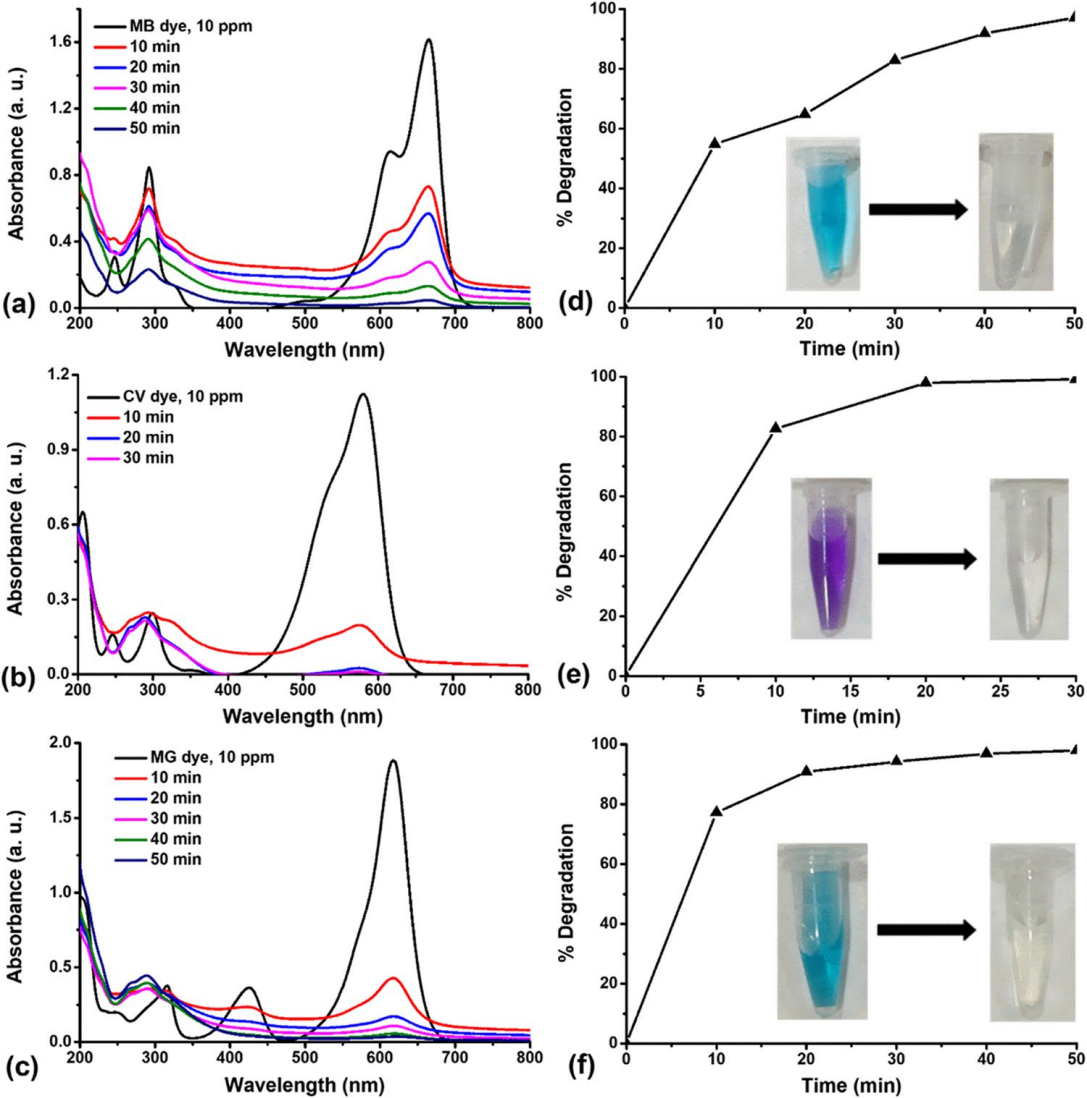
Absorption spectra of photocatalytic degradation of (**a**) MB, (**b**) CV, and (**c**) MG dye treated with Au@Fe_3_O_4_ BNPs at different time points and degradation percentages of (**d**) MB, (**e**) CV, and (**f**) MG dye

**Fig. 10:**
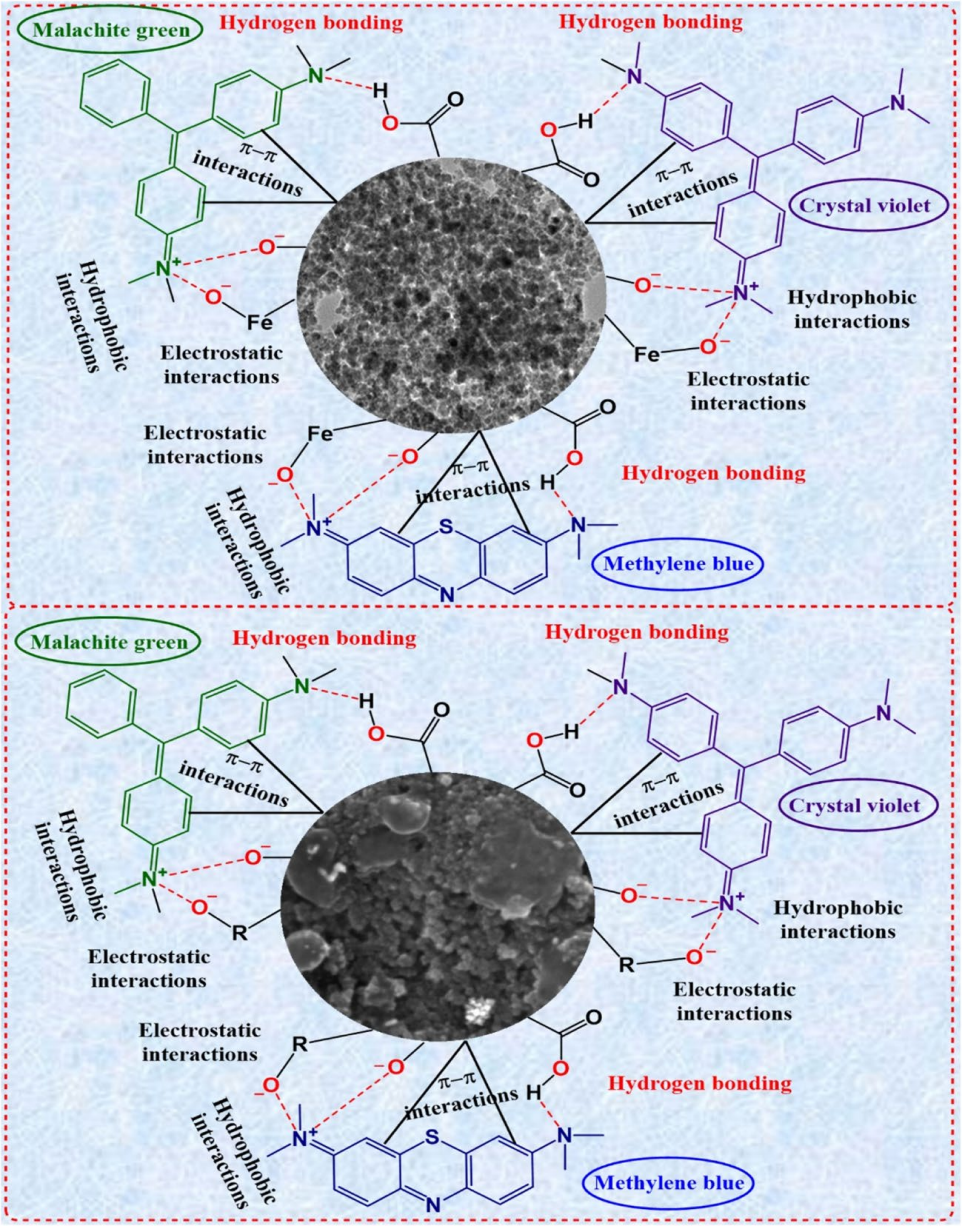
The adsorption mechanism of MB, CV, MG onto (**a**) Fe_3_O_4_ NPs and (**b**) Au@Fe_3_O_4_ NPs

High efficiency was observed in the removal of textile-toxic basic dyes (MB, CV, and MG dye) using biosynthesized Au@Fe_3_O_4_ BNPs as nanocatalyst at room temperature, without the addition of any catalyst. Figure 9 represents UV-visible absorption plots of photocatalytic degradation of MB, CV, and MG dye treated with Au@Fe_3_O_4_ BNPs at different time intervals and their degradation percentages. Increasing the contact time resulted in a higher percentage of dye removal for all dyes examined. The ideal contact times for removing MB, CV, and MG were determined to be 50 min, 30 min, and 50 min, respectively, leading to the highest levels of dye removal (Fig. 9a, b, c). As displayed in Fig. 9d, e, f, Au@Fe_3_O_4_ BNPs degraded the MB, CV and MG dye at a concentration of 10 ppm with 97.1%, 99.1%, and 98.1% degradation efficiency, respectively. Meanwhile, the removal of MB, CV, and MG dyes using Au@Fe_3_O_4_ NPs was performed in the dark for 50 min, 30 min, and 50 min, respectively, as shown in Fig. S8. Still, the removal percentage efficiency was relatively lesser compared to the degradation efficiency of MB, CV, and MG dye in the presence of light (Table S7). The removal efficiency time for the dark reaction was 20 h for MB, CV, and MG dyes, whereas the degradation efficiency time for the light reaction was 50 min, 30 min, and 50 min for MB, CV, and MG dyes, respectively. This improvement in dye removal percentage with extended contact time can be attributed to the stronger attraction between dye molecules and the active sites on the adsorbent molecules. The effectiveness of Au@Fe_3_O_4_ BNPs synthesized through the green method can be attributed to the presence of elemental gold and biomolecule compounds from propolis extract, forming a coating on the Fe_3_O_4_ NPs surface. Furthermore, the core-shell structure and the larger surface area of these NPs contribute significantly to their high adsorption capacity for cationic dyes (Das et al. 2023). It appears that the functional groups on the nanoparticle surface play a crucial role in the adsorption process, as indicated by FTIR and XPS analysis. These functional groups can potentially interact with the functional groups of dyes (MB, CV, MG) through hydrogen bonding. These functional groups can potentially interact with the functional groups of dyes (MB, CV, MG) through the presence of certain forces, such as hydrogen bonding, electrostatic interactions, and hydrophobic-hydrophobic interactions (Fig. 10b). There is no significant difference between the adsorption capacities of Fe_3_O_4_ and Au@Fe_3_O_4_ in the degradation of those dyes. In summary, the results demonstrate that biosynthesized Fe_3_O_4_ NPs and Au@Fe_3_O_4_ BNPs are effective in adsorbing basic dyes with cationic charged surfaces. Table S8 shows a comparative table of the photocatalytic activity of Fe_3_O_4_ NPs and Au@Fe_3_O_4_ BNPs with some reported materials.

To check the reusability of Au@Fe_3_O_4_ NPs, FTIR analysis was conducted for both fresh and regenerated Au@Fe_3_O_4_ NPs. The desorption of the synthesized NPs involved washing them three times with ethanol and subsequently drying them in the oven. According to the FTIR results depicted in Fig. S9, there were no significant changes in the nature of the nanoparticles, confirming the stability of the regenerated Au@Fe_3_O_4_. Therefore, these green synthesized stable nanoparticles can be effectively utilized for the removal of toxic dyes from aqueous solutions.

#### FTIR analysis of MB, CV, and MG after adsorption

**Fig. 11:**
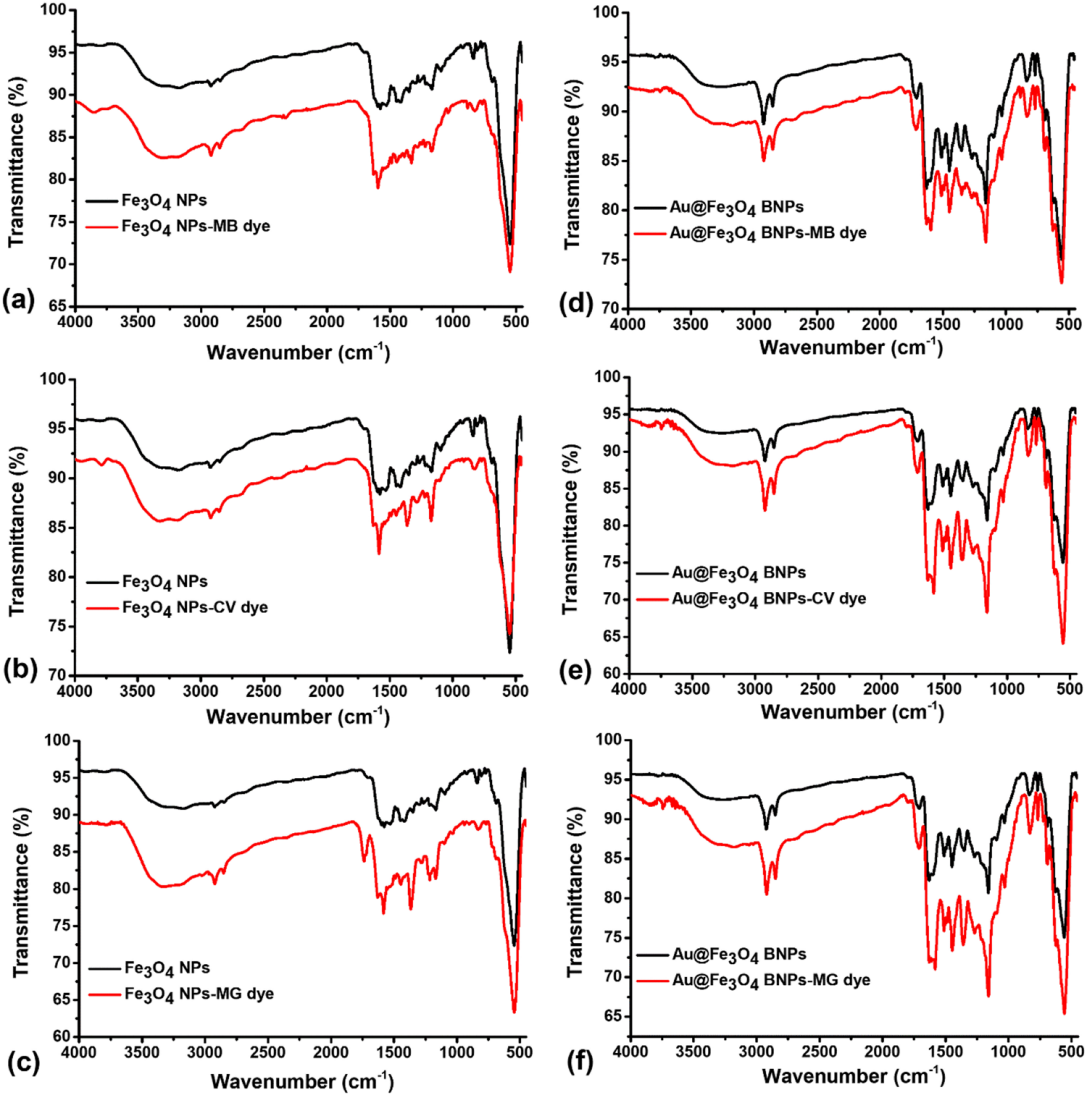
FTIR spectra of Fe_3_O_4_ NPs before adsorption of MB/CV/MG dyes, (**a**) after MB adsorption, (**b**) after CV adsorption, and (**c**) after MG adsorption; FTIR spectra of Au@Fe_3_O_4_ BNPs before adsorption of MB/CV/MG dyes, (**d**) after MB adsorption, (**e**) after CV adsorption, and (**f**) after MG adsorption

After the MB, CV, and MG dye adsorption processes using Fe_3_O_4_ NPs and Au@Fe_3_O_4_ BNPs, the intensity of the peaks in the adsorbents shifted without any change in their positions (Fig. 11). This shift can be attributed to the interaction and binding of the MB, CV, and MG dyes with the adsorbent surface. In the FTIR spectrum of Fe_3_O_4_ NPs-MB and Au@Fe_3_O_4_ BNPs-MB, the peaks corresponding to the -OH, C=O, C=C, and C-O-C functional groups shifted to lower wavelengths, indicating adsorptive interactions between these functional groups present on the surfaces of Fe_3_O_4_ NPs and Au@Fe_3_O_4_ BNPs with the MB dye (Matar and Andac 2023).

In the FTIR spectra of Fe_3_O_4_ NPs-CV and Au@Fe_3_O_4_ BNPs-CV, important sharp peaks appeared at 1584 cm^-1^ and 1361 cm^-1^ for Fe_3_O_4_ NPs-CV, and at 1584 cm^-1^ and 1360 cm^-1^ for Au@Fe_3_O_4_ BNPs-CV. These peaks are attributed to the C=C aromatic stretching vibration and C–N aromatic tertiary amine stretching vibration, respectively (Foroutan et al. 2021; Shalaby et al. 2021). Distinctive changes were observed in the FTIR spectrum of Fe_3_O_4_ NPs-MG and Au@Fe_3_O_4_ BNPs-MG. A new sharp peak at 1735 cm^-1^ appeared in the FTIR spectrum of Fe_3_O_4_ NPs-MB, corresponding to the C=O stretching vibration. The other peaks at 1583 cm^-1^ and 1365 cm^-1^ are associated with C=C stretching in the benzene rings and C–N stretching vibration, respectively. Similarly, in the FTIR Spectrum of Au@Fe_3_O_4_ BNPs-MG, the peaks of the -OH, C=O, C=C, and C-O-C functional groups also shifted to lower wavelengths, along with the C–N stretching vibration (Zhang et al. 2022). These observations confirm the adsorption interaction of the functional groups on the surfaces of Fe_3_O_4_ NPs and Au@Fe_3_O_4_ BNPs with MB, CV, and MG.

### Study of photodegradation kinetics

The Fe_3_O_4_ NPs and Au@Fe_3_O_4_ BNPs conducted kinetic investigations on the photodegradation of MB, CV, and MG dyes using the pseudo-first-order and pseudo-second-order models (Purkait et al. 2023). These models assist in determining the rate constants for the photodegradation of MB, CV, and MG dyes in the presence of Fe_3_O_4_ NPs and Au@Fe_3_O_4_ BNPs as photocatalysts, and the rate constants obtained were used to understand the mechanism of the reaction, as indicated by the following equations (6-7):

6 $$\ln {\textrm{A}}_{\textrm{t}}=-{\textrm{K}}_1\textrm{t}+\ln {\textrm{A}}_{{}^{\circ}}$$

7 $$\frac{1}{{\textrm{A}}_{\textrm{t}}}={\textrm{K}}_2\textrm{t}+\frac{1}{{\textrm{A}}_{{}^{\circ}}}$$

where K_1_ and K_2_ are the adsorption rate constants of the Langmuir-Hinshelwood model and the pseudo-second-order model. Once the suitable linear graphs for these models were plotted, we proceeded to compute the constant values, which are presented in Table 1.

**Table 1:** Kinetics parameters associated with the photocatalytic degradation of MB, CV, and MG dye using Fe_3_O_4_ NPs and Au@Fe_3_O_4_ BNPs

Samples	First-order kinetic model		Second-order kinetic model		degradation (%)
	K_1_ (min^-1^)	R^2^	K_2_ (L mg^-1^ min^-1^)	R^2^	
MB-Fe_3_O_4_ NPs	0.0430	0.9938	0.1736	0.8454	95.24
CV-Fe_3_O_4_ NPs	0.0969	0.9730	2.2191	0.5788	99.37
MG-Fe_3_O_4_ NPs	0.0646	0.9466	0.2263	0.9867	96.23
MB-Au@Fe_3_O_4_ BNPs	0.0652	0.9892	0.3536	0.7059	97.09
CV-Au@Fe_3_O_4_ BNPs	0.1686	0.9886	3.3387	0.8832	99.11
MG-Au@Fe_3_O_4_ BNPs	0.0881	0.9717	0.5187	0.9192	98.04

**Fig. 12:**
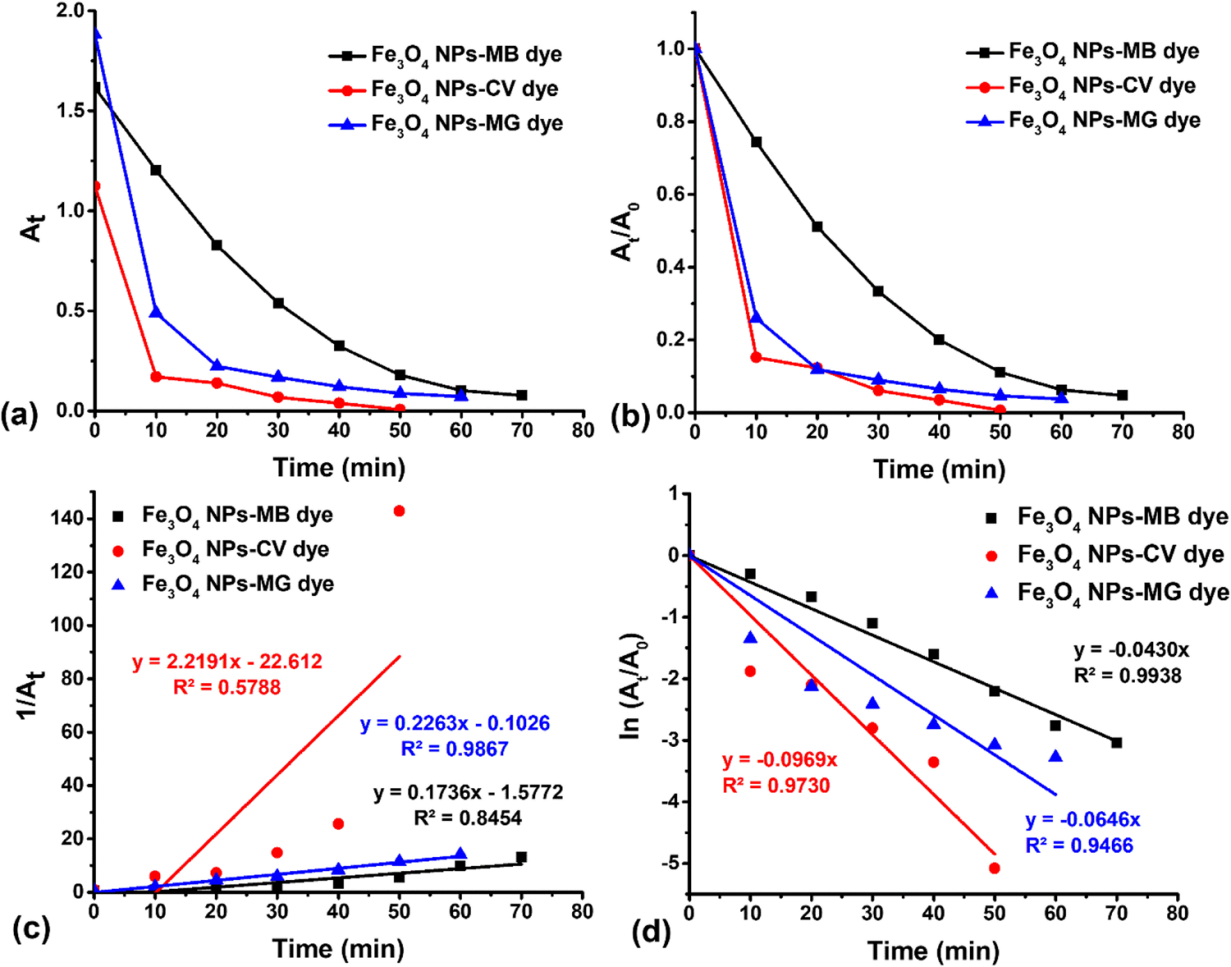
(**a**) Variation of absorbance (At) of MB, CV, and MG dye degradation with time; (**b**) pseudo-second-order kinetics of MB, CV, and MG dye degradation by Fe_3_O_4_ NPs; (**c**) Variation of absorbance (1/At) of MB, CV, and MG dye degradation with time; (**d**) pseudo-first-order kinetics of MB, CV, and MG dye degradation by Fe_3_O_4_ NPs

Fig. 12 illustrates kinetic graphs representing both the pseudo-first-order model and pseudo-second-order kinetic models of MB-Fe_3_O_4_ NPs, CV-Fe_3_O_4_ NPs, and MG-Fe_3_O_4_ NPs. Table 1 presents a summary of the correlation coefficient (R^2^) values and various rate constants. The pseudo-first-order kinetics values of K_1_ and R^2^ for the MB-Fe_3_O_4_ NPs, CV-Fe_3_O_4_ NPs, and MG-Fe_3_O_4_ NPs are found to be (0.0430 min^−1^ and 0.9938), (0.0969 min^−1^ and 0.9730) and (0.0646 min^−1^ and 0.9466), respectively. In the case of pseudo-second-order kinetics values of K_2_ and R^2^ for the MB-Fe_3_O_4_ NPs, CV-Fe_3_O_4_ NPs, and MG-Fe_3_O_4_ NPs are found to be (0.1736 min^−1^ and 0.8454), (2.2191 min^−1^ and 0.5788) and (0.2263 min^−1^ and 0.9867), respectively. It's evident that, in terms of contact time, the pseudo-first-order kinetics proved to be the most favorable for degrading MB, CV, and MG using Fe_3_O_4_ NPs. Their respective R^2^ values were notably high at 0.9908, 0.9336, and 0.8834, respectively.

**Fig. 13:**
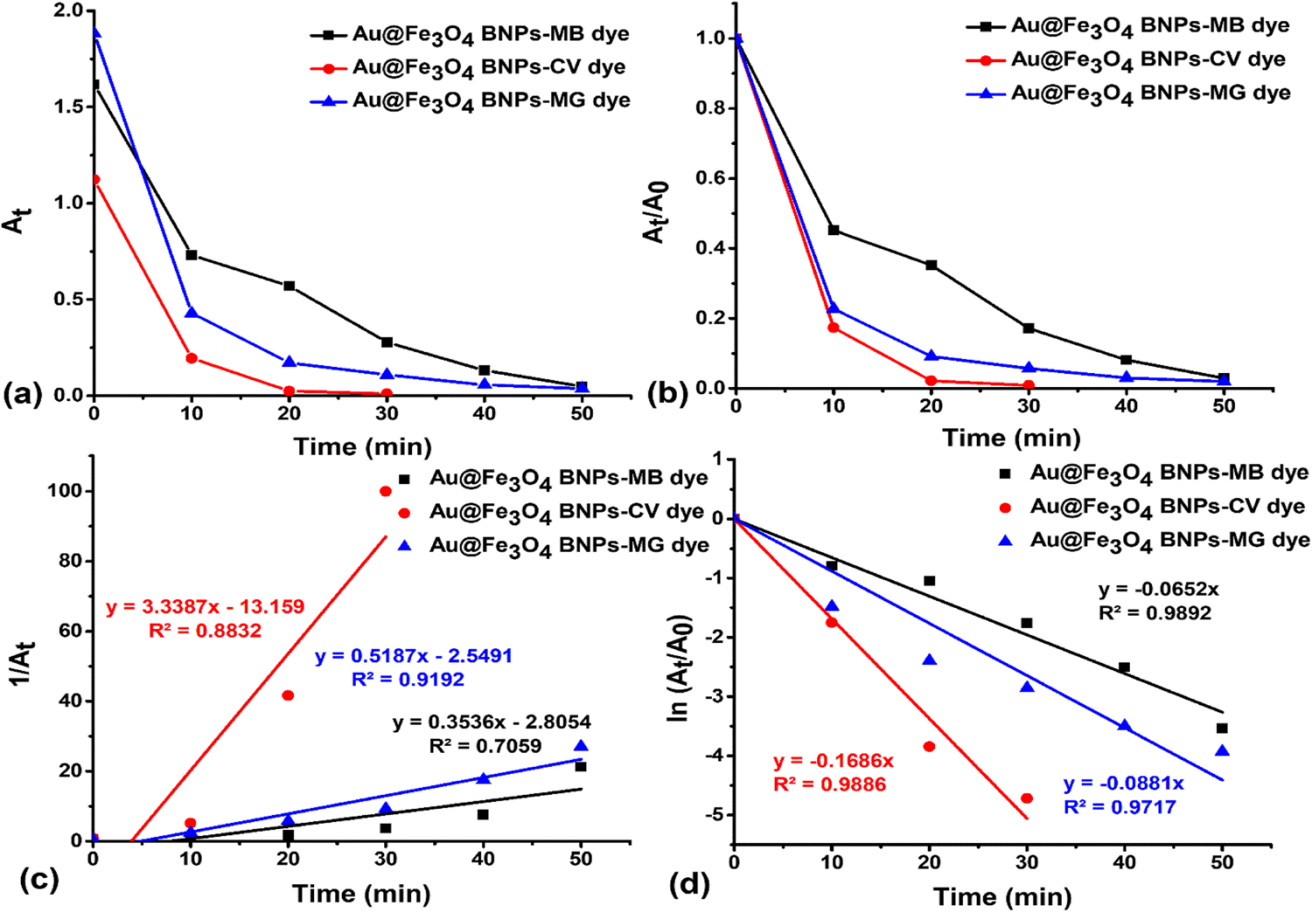
(**a**) Variation of absorbance (At) of MB, CV, and MG dye degradation with time; (**b**) pseudo-second-order kinetics of MB, CV, and MG dye degradation by Au@Fe_3_O_4_ BNPs; (**c**) Variation of absorbance (1/At) of MB, CV, and MG dye degradation with time; (**d**) pseudo-first-order kinetics of MB, CV, and MG dye degradation by Au@Fe_3_O_4_ BNPs

Fig. 13 illustrates kinetic graphs representing both the pseudo-first-order model and pseudo-second-order kinetic models of MB-Au@Fe_3_O_4_ BNPs, CV-Au@Fe_3_O_4_ BNPs, and MG-Au@Fe_3_O_4_ BNPs. Table 1 presents a summary of the correlation coefficient (R^2^) values and various rate constants. The pseudo-first-order kinetics values of K_1_ and R^2^ for the MB-Au@Fe_3_O_4_ BNPs, CV-Au@Fe_3_O_4_ BNPs, and MG-Au@Fe_3_O_4_ BNPs are found to be (0.0652 min^−1^ and 0.9892), (0.1686 min^−1^ and 0.9886) and (0.0881 min^−1^ and 0.9717), respectively. In the case of pseudo-second-order kinetics values of K_2_ and R^2^ for the MB-Au@Fe_3_O_4_ BNPs, CV-Au@Fe_3_O_4_ BNPs, and MG-Au@Fe_3_O_4_ BNPs are found to be (0.3536 min^−1^ and 0.7059), (3.3387 min^−1^ and 0.8832) and (0.5187 min^−1^ and 0.9192), respectively. It's evident that, in terms of contact time, the pseudo-first-order kinetics proved to be the most favorable for degrading MB, CV, and MG using Au@Fe_3_O_4_ BNPs, with corresponding R^2^ values of 0.9751, 0.9768, and 0.9448, respectively.

### DPPH radical scavenging activity

**Fig. 14:**
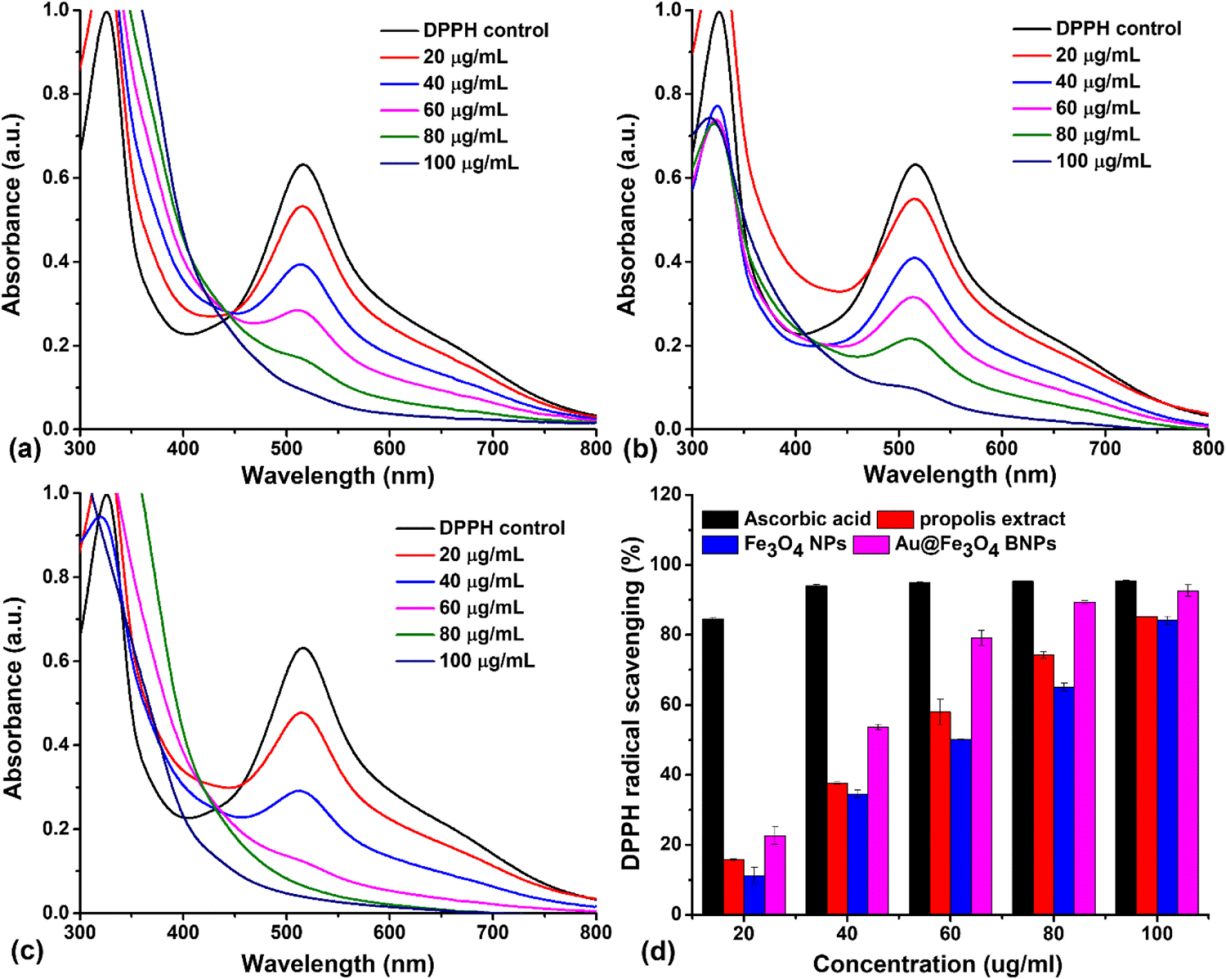
UV-visible spectrum of DPPH^•^ scavenging using (**a**) propolis extract, (**b**) Fe_3_O_4_ NPs and (**c**) Au@Fe_3_O_4_ BNPs; (**d**) Percentage inhibition of DPPH^•^ using ascorbic acid (as standard), propolis extract, Fe_3_O_4_ NPs and Au@Fe_3_O_4_ BNPs. Statistical analysis: the values are presented as mean ± standard deviation

The DPPH radical scavenging activity of propolis extract, Fe_3_O_4_ NPs, and Au@Fe_3_O_4_ BNPs was evaluated. In an ethanolic solution of DPPH, there exists a DPPH^•^ free radical, which readily accepts either an electron or hydrogen to transform into a stable diamagnetic molecule known as DPPH-H (Hertadi et al. 2020). The introduction of propolis extract, Fe_3_O_4_ NPs and Au@Fe_3_O_4_ BNPs caused the donation of electrons to DPPH^•^ free radicals, resulting in the conversion of the purple-colored free radicals into a pale yellowish DPPH-H form. Figure 14 represents UV-visible absorption plots of DPPH^•^ scavenging using propolis extract, Fe_3_O_4_ NPs, and Au@Fe_3_O_4_ BNPs at various concentrations (20, 40, 60, 80, and 100 μg/mL), along with their respective percentages of inhibition of DPPH^•^. Consequently, there was a reduction in absorbance observed at 517 nm as the concentration of propolis extract, Fe_3_O_4_ NPs and Au@Fe_3_O_4_ BNPs increased. The antioxidant capacity of propolis extract, Fe_3_O_4_ NPs and Au@Fe_3_O_4_ BNPs exhibited an upward trend with increasing concentration, as depicted in Fig. 14d. Within the concentration range of 20–100 μg/mL, the inhibition of DPPH^•^ free radicals increased from 15.8% to 85.2% for propolis extract, 11.2% to 84.2% for Fe_3_O_4_ NPs, and 22.7% to 92.6% for Au@Fe_3_O_4_ BNPs, compared to the standard ascorbic acid (which exhibited inhibition rates between 84.5% and 95.4%). The IC_50_ values for the propolis extract, Fe_3_O_4_ NPs and Au@Fe_3_O_4_ BNPs in the DPPH radical scavenging method were determined to be 55.23 ± 0.98 μg/ml, 61.09 ± 1.31 μg/ml, and 40.08 ± 1.94 μg/ml, respectively. Among them, Au@Fe_3_O_4_ BNPs exhibited the most potent antioxidant activity, as indicated by the lowest IC_50_ value in the DPPH assay.

## Conclusion

A sustainable and cost-efficient approach has been utilized to produce environmentally friendly adsorbents as highly functional magnetic bimetallic NPs. Specifically, Fe_3_O_4_ NPs and Au@Fe_3_O_4_ BNPs were synthesized using propolis extract as both a reducing and capping agent. The UV-visible absorption spectra confirmed the presence of characteristic absorption peaks and the successful formation of these NPs. The FTIR analysis provided insights into the functional groups responsible for reducing and stabilizing the NPs. XRD analysis revealed the nature crystallinity of both Fe_3_O_4_ NPs and Au@Fe_3_O_4_ BNPs, with characteristic diffraction peaks matching their respective crystal structures. The VSM measurements demonstrated the supermagnetic nature of these NPs. Morphological analysis through FE-SEM and TEM showed that Fe_3_O_4_ NPs had uniform spherical shapes with an average size of 5.45 ± 1.28 nm, whereas Au@Fe_3_O_4_ BNPs exhibited various shapes and aggregated structures with an average size of 15.67 ± 4.02 nm. XPS analysis provided insights into the oxidation states and surface chemistry of Fe_3_O_4_ NPs and Au@Fe_3_O_4_ BNPs, further confirming the successful synthesis and the presence of functional groups on their surfaces. The photocatalytic performance of Fe_3_O_4_ NPs and Au@Fe_3_O_4_ BNPs in degrading cationic dyes (MB, CV, MG) was highly efficient, with Fe_3_O_4_ NPs exhibiting excellent dye removal percentages: 95.2% in 70 min for MB, 99.4% in 50 min for CV, and 96.2% in 60 min for MG. Au@Fe_3_O_4_ BNPs, with their unique surface properties, also demonstrated dye removal percentages: 97.1% in 50 min for MB, 99.1% in 50 min for CV, and 98.1% in 60 min for MG. The kinetic analysis revealed that the pseudo-first-order kinetics model was the most suitable for describing the dye degradation process. Moreover, the DPPH radical scavenging assay demonstrated the antioxidant potential of propolis extract, Fe_3_O_4_ NPs, and Au@Fe_3_O_4_ BNPs; among these, Au@Fe_3_O_4_ BNPs exhibited the highest antioxidant activity. These newly synthesized NPs hold promise for a wide range of applications, including wastewater treatment, catalysis, and biomedical fields, owing to their magnetic, catalytic, and antioxidant properties.

## Supplementary Information

ESM 1: Supplementary Figures and Tables (DOCX 2529 kb)

## Data Availability

Not applicable.
