# Supplementary material for: Highly efficient degradation of basic dyes using gold-coated nature-based supermagnetic iron oxide nanoparticles as eco-friendly nanocatalysts
Source: Environ Sci Pollut Res Int. 2024 Mar 9;31(17):24894–912. doi: 10.1007/s11356-024-32775-3 (PMC11636711; doi:10.1007/s11356-024-32775-3)
Supplement: Supplementary file 1 — Supplementary Figures and Tables (DOCX 2529 kb) [file 11356_2024_32775_MOESM1_ESM.docx]

**Highly efficient degradation of basic dyes using gold-coated nature-based supermagnetic iron oxide nanoparticles as eco-friendly nanocatalysts**

**Ghassan H. Matar^*a^, Muberra Andac^a, b^**

^a^Department of Chemistry, Ondokuz Mayis University, Samsun, Turkey

^b^Department of Nanoscience and Nanotechnology, Ondokuz Mayis University, Samsun, Turkey

*Corresponding author at.

Department of Chemistry, Ondokuz Mayis University, Samsun, Turkey.

E-mail addresses: ghassanhaz8@gmail.com (Ghassan H. Matar)

**Supplementary Material**

**SUPPLEMENTARY MATERIAL**


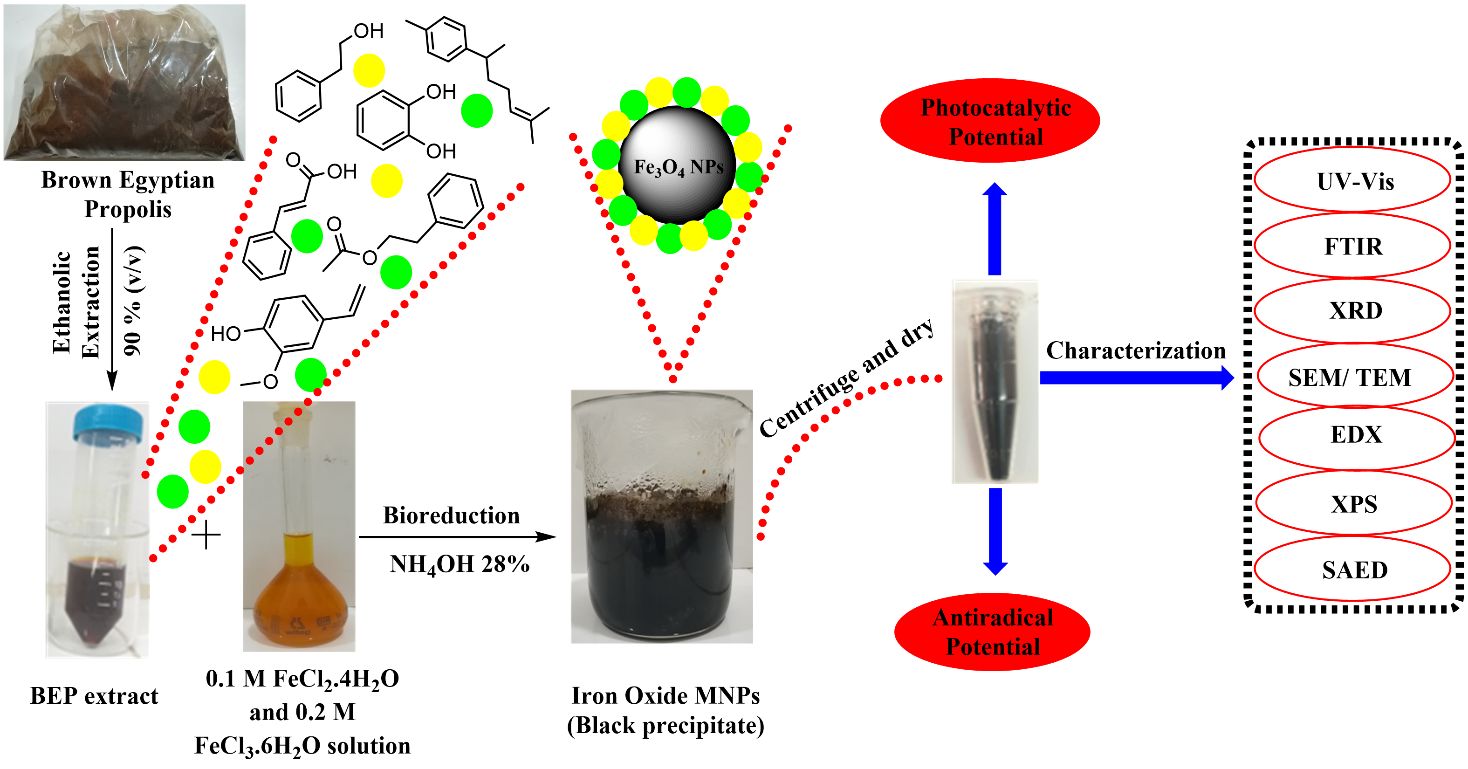


Fig. S1. Schematic of superparamagnetic Fe_3_O_4_ NPs green synthesis steps using propolis extract.

Table S1. Calculation of average crystallite size AuNPs, Fe_3_O_4_ NPs and Au@Fe_3_O_4_ BNPs using XRD (Debye-Scherrer equation)

|  | 2 θ in degree | FWHM in degree | θ in radians | FWHM in radians | hkl | d value (Å) | Size (nm) | Average size (nm) |
| --- | --- | --- | --- | --- | --- | --- | --- | --- |
| AuNPs | 38.147 | 0.878 | 0.3328 | 0.0153 | (111) | 2.3572 | 9.63 | 8.59 |
|  | 44.417 | 1.342 | 0.3876 | 0.0234 | (200) | 2.0379 | 6.42 |  |
|  | 64.671 | 1.012 | 0.5643 | 0.0176 | (220) | 1.4401 | 9.37 |  |
|  | 77.433 | 1.141 | 0.6757 | 0.0199 | (311) | 1.2315 | 8.94 |  |
| Fe_3_O_4_ NPs | 30.410 | 0.680 | 0.2653 | 0.0118 | (220) | 2.9370 | 12.17 | 11.66 |
|  | 35.490 | 0.690 | 0.3097 | 0.012 | (311) | 2.5277 | 12.13 |  |
|  | 43.414 | 0.769 | 0.3788 | 0.0134 | (400) | 2.0826 | 11.13 |  |
|  | 54.005 | 0.769 | 0.4713 | 0.0134 | (422) | 1.6965 | 11.61 |  |
|  | 57.180 | 0.770 | 0.4990 | 0.0134 | (511) | 1.6098 | 11.78 |  |
|  | 62.880 | 0.840 | 0.5487 | 0.0146 | (440) | 1.4768 | 11.13 |  |
| Au@Fe_3_O_4_ BNPs | 30.161 | 0.689 | 0.2632 | 0.0120 | (220)* | 2.9606 | 11.97 | 14.28 |
|  | 35.572 | 0.716 | 0.3104 | 0.0125 | (311)* | 2.5218 | 11.65 |  |
|  | 38.156 | 0.411 | 0.3329 | 0.0071 | (111)° | 2.3567 | 20.66 |  |
|  | 43.244 | 0.689 | 0.3773 | 0.0120 | (400)* | 2.0904 | 12.43 |  |
|  | 44.377 | 0.689 | 0.3872 | 0.0120 | (200)° | 2.0396 | 12.48 |  |
|  | 53.778 | 0.689 | 0.4693 | 0.0120 | (422)* | 1.7031 | 12.95 |  |
|  | 57.220 | 0.720 | 0.4993 | 0.0125 | (511)* | 1.6086 | 12.63 |  |
|  | 62.780 | 0.790 | 0.5478 | 0.0137 | (440)* | 1.4789 | 11.85 |  |
|  | 64.580 | 0.560 | 0.5635 | 0.0097 | (220)° | 1.4420 | 16.91 |  |
|  | 77.424 | 0.530 | 0.6756 | 0.0092 | (311)° | 1.2733 | 19.31 |  |

* Fe_3_O_4_ NPs ° AuNPs

Table S2. calculation of average crystallite size AuNPs, Fe_3_O_4_ NPs and Au@Fe_3_O_4_ BNPs using XRD (Williamson-Hall (W-H) equation)

|  | 4 sinθ | βcosθ | intercept | slope | Average size (nm) |
| --- | --- | --- | --- | --- | --- |
| AuNPs | 1.30712 | 0.01448 | 0.02022 | -0.0019 | 6.8 |
|  | 1.51191 | 0.02168 |  |  |  |
|  | 2.13950 | 0.01492 |  |  |  |
|  | 2.50187 | 0.01554 |  |  |  |
| Fe3O4 NPs | 1.0488 | 0.01139 | 0.01071 | 0.00075 | 12.9 |
|  | 1.21909 | 0.01143 |  |  |  |
|  | 1.47922 | 0.01245 |  |  |  |
|  | 1.81618 | 0.01194 |  |  |  |
|  | 1.91419 | 0.01177 |  |  |  |
|  | 2.08631 | 0.01246 |  |  |  |
| Au@Fe3O4 BNPs | 1.04073 | 0.01162 | 0.0129 | -0.001 | 10.7 |
|  | 1.22185 | 0.0119 |  |  |  |
|  | 1.30742 | 0.00678 |  |  |  |
|  | 1.47394 | 0.01119 |  |  |  |
|  | 1.51062 | 0.01114 |  |  |  |
|  | 1.80906 | 0.01073 |  |  |  |
|  | 1.91538 | 0.01103 |  |  |  |
|  | 2.08344 | 0.01177 |  |  |  |
|  | 2.13682 | 0.00826 |  |  |  |
|  | 2.50162 | 0.00722 |  |  |  |

|  | FWHM/tanθsinθ | (FWHM/tanθ)^2^ | intercept | slope | R^2^ | D_XRD_ (nm) |
| --- | --- | --- | --- | --- | --- | --- |
| AuNPs | 0.13562 | 0.00196 | -2.831 × 10^-4^ | 0.02052 | 0.8357 | 6.7 |
|  | 0.15178 | 0.00329 |  |  |  |  |
|  | 0.05216 | 0.00078 |  |  |  |  |
|  | 0.03972 | 0.00062 |  |  |  |  |
| Fe_3_O_4_ NPs | 0.16650 | 0.00191 | 5.903 × 10^-5^ | 0.01115 | 0.9956 | 12.4 |
|  | 0.12348 | 0.00142 |  |  |  |  |
|  | 0.09116 | 0.00114 |  |  |  |  |
|  | 0.05801 | 0.00069 |  |  |  |  |
|  | 0.05153 | 0.00061 |  |  |  |  |
|  | 0.04597 | 0.00057 |  |  |  |  |
| Au@Fe_3_O_4_ BNPs | 0.17167 | 0.00200 | -1.084 × 10^-4^ | 0.01231 | 0.9732 | 11.2 |
|  | 0.12753 | 0.00152 |  |  |  |  |
|  | 0.06346 | 0.00043 |  |  |  |  |
|  | 0.08240 | 0.00092 |  |  |  |  |
|  | 0.07814 | 0.00087 |  |  |  |  |
|  | 0.05248 | 0.00056 |  |  |  |  |
|  | 0.04811 | 0.00053 |  |  |  |  |
|  | 0.04338 | 0.00051 |  |  |  |  |
|  | 0.02895 | 0.00024 |  |  |  |  |
|  | 0.01845 | 0.00013 |  |  |  |  |

Table S3. Calculation of average crystallite size AuNPs, Fe3O4 NPs and Au@Fe3O4 BNPs using XRD (Halder-Wagner (H-M) equation)







Fig. S2. Williamson-Hall (W-H) plots of (a) biosynthesized Fe_3_O_4_ NPs, (b) Au@Fe_3_O_4_ BNPs and (e) AuNPs; Halder-Wagner (H-W) plots of (c) biosynthesized Fe_3_O_4_ NPs, (d) Au@Fe_3_O_4_ BNPs and (f) AuNPs.

Table S4. Values of different magnetization parameters obtained by VSM analysis.

| NPs | Magnetization (Ms) (emu/g) | Coercivity  jHc (Oe) | Saturation remanence (Mr) (emu/g) |
| --- | --- | --- | --- |
| Fe_3_O_4_ NPs | 57.22 | 37.13 | 4.71 |
| Au@Fe_3_O4 NPs | 30.99 | 39.65 | 2.80 |

Table S5. calculation of d-spacing (Å) for Fe_3_O_4_ NPs and Au@Fe_3_O_4_ BNPs using SAED pattern.

| Sample | 1/2r (1/nm) | 1/r (1/nm) | r (nm) | d-spacing (Å) | hkl |
| --- | --- | --- | --- | --- | --- |
| Fe_3_O_4_ NPs | 6.825 | 3.413 | 0.2930 | 2.930 | (220) |
|  | 7.930 | 3.965 | 0.2522 | 2.522 | (311) |
|  | 9.737 | 4.869 | 0.2054 | 2.054 | (400) |
|  | 11.780 | 5.890 | 0.1698 | 1.698 | (422) |
|  | 12.463 | 6.232 | 0.1605 | 1.605 | (511) |
|  | 13.640 | 6.820 | 0.1466 | 1.466 | (440) |
| Au@Fe_3_O_4_ BNPs | 6.764 | 3.382 | 0.2956 | 2.956 | (220)* |
|  | 7.941 | 3.970 | 0.2518 | 2.518 | (311)* |
|  | 8.450 | 4.225 | 0.2366 | 2.366 | (111)° |
|  | 9.641 | 4.820 | 0.2074 | 2.074 | (400)* |
|  | 9.860 | 4.930 | 0.2028 | 2.028 | (200)° |
|  | 11.469 | 5.734 | 0.1743 | 1.743 | (422)* |
|  | 12.353 | 6.176 | 0.1619 | 1.619 | (511)* |
|  | 13.455 | 6.727 | 0.1486 | 1.486 | (440)* |
|  | 13.970 | 6.985 | 0.1431 | 1.431 | (220)° |
|  | 15.878 | 7.939 | 0.1259 | 1.259 | (311)° |

*Fe_3_O_4_ NPs °AuNPs

Fig. S3. Zeta potential of (a) the Fe_3_O_4_ NPs and (b) Au@Fe_3_O_4_ NPs.





Fig. S4. (a) XPS survey spectrum of Fe_3_O_4_ NPs, (b) Fe2p spectra, (c) C 1s spectra, and (d) O 1s spectra.

Table S6. Detailed information of elemental composition from XPS analysis.

| Sample Name | Element Name | Peak BE | FWHM (eV) | Area (CPS. eV) | Atomic % |
| --- | --- | --- | --- | --- | --- |
| Fe_3_O_4_ NPs | C 1s | 285.15 | 2.86 | 262640.73 | 36.08 |
|  | O 1s | 530.52 | 1.65 | 682889.59 | 38.76 |
|  | Fe 2p | 711.20 | 4.21 | 955674.93 | 12.73 |
| Au@Fe_3_O_4_ BNPs | C 1s | 284.37 | 1.51 | 63500.97 | 51.50 |
|  | O 1s | 532.64 | 2.30 | 53310.22 | 25.50 |
|  | Fe 2p | 710.94 | 1.88 | 3980.23 | 13.5 |
|  | Au 4f | 84.13 | 1.25 | 4053.40 | 7.5 |


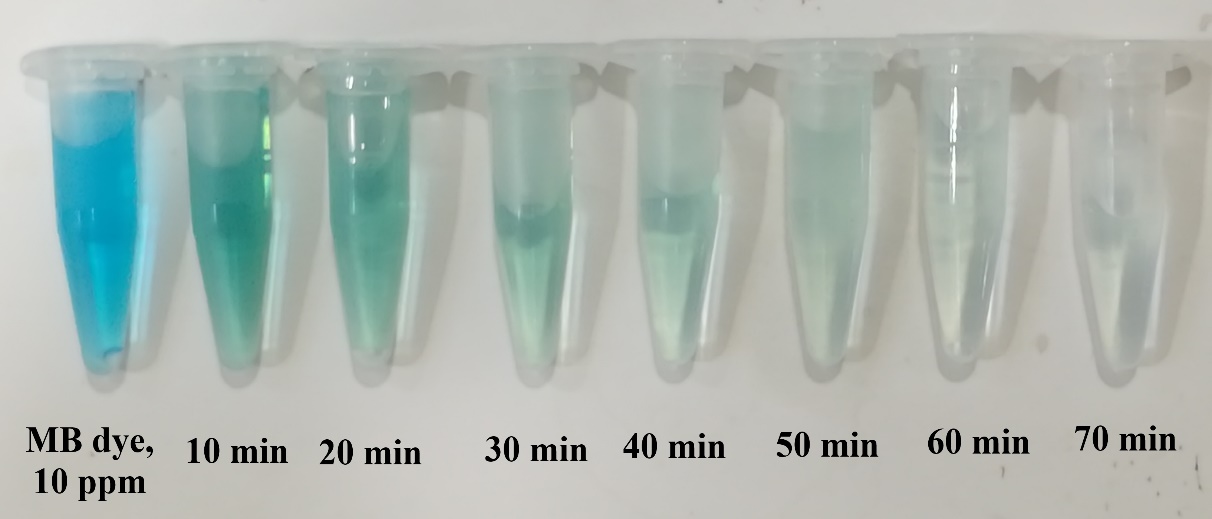


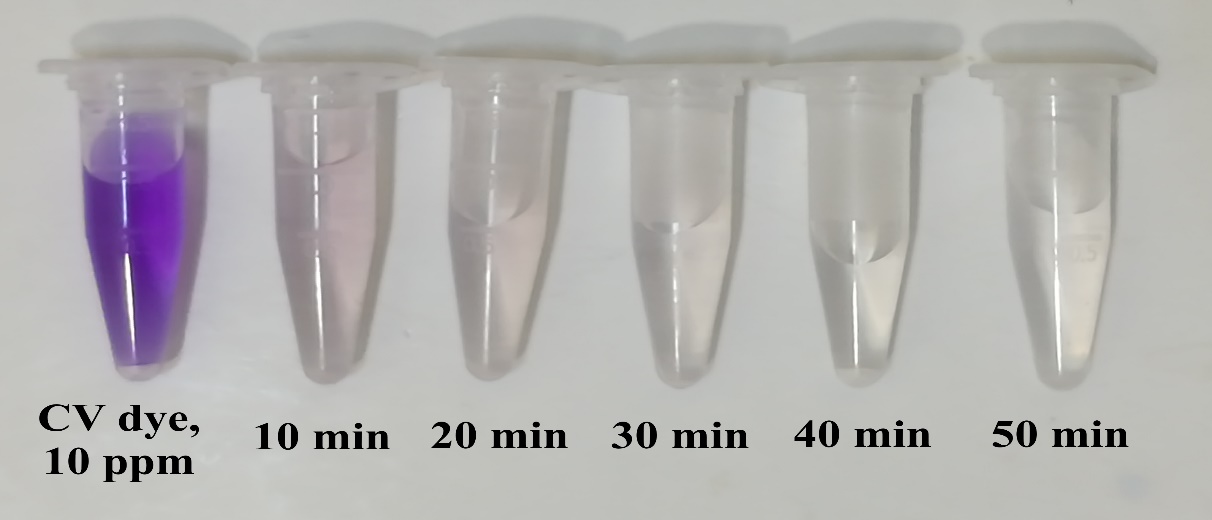


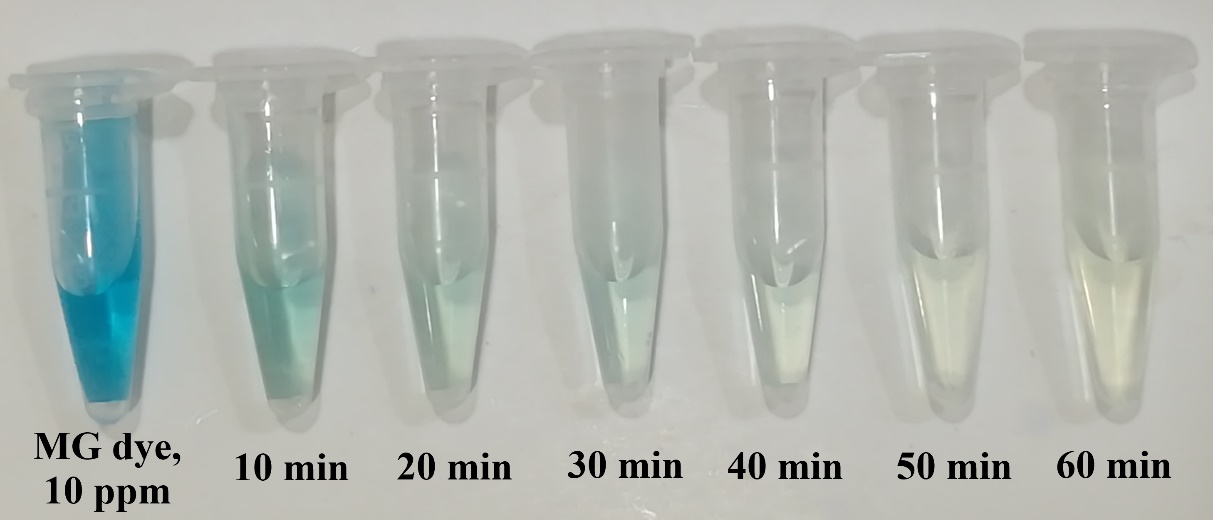


Fig. S5. Representation of the gradual degradation of MB, CV, and MG dye (color change) in the presence of biosynthesized Fe_3_O_4_ NPs.


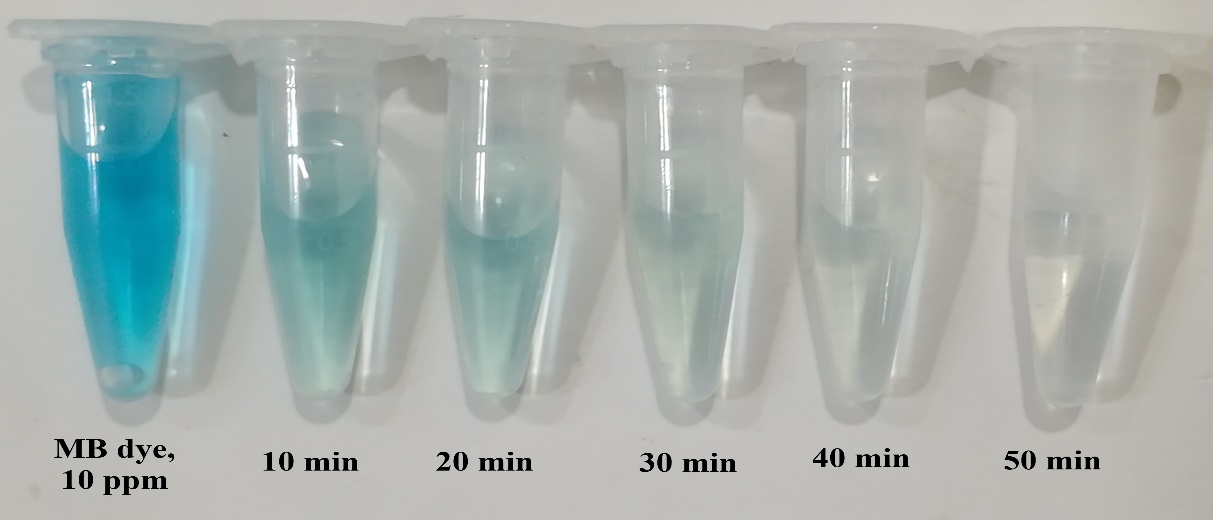


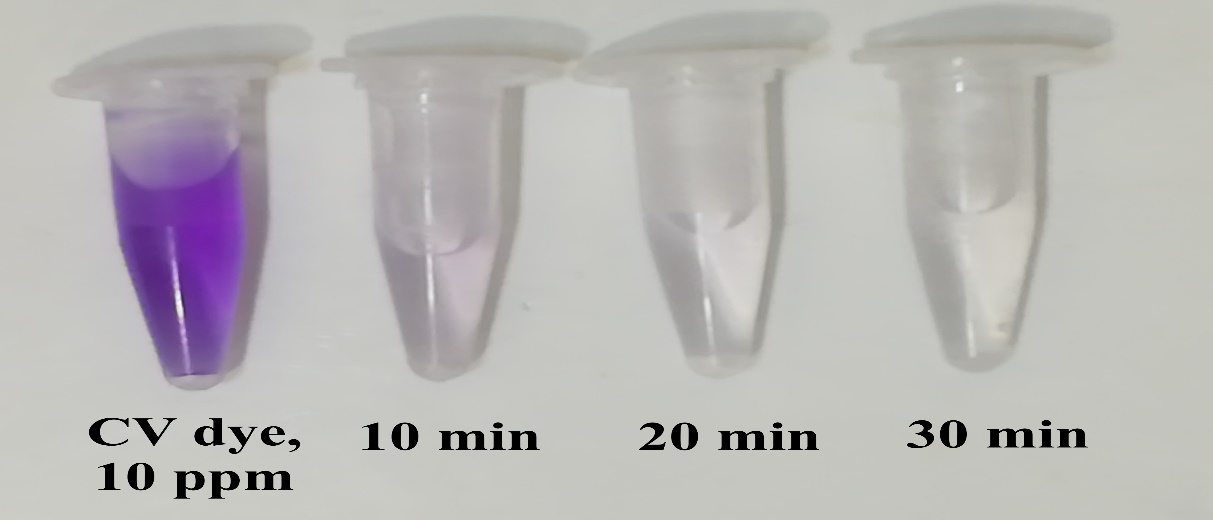


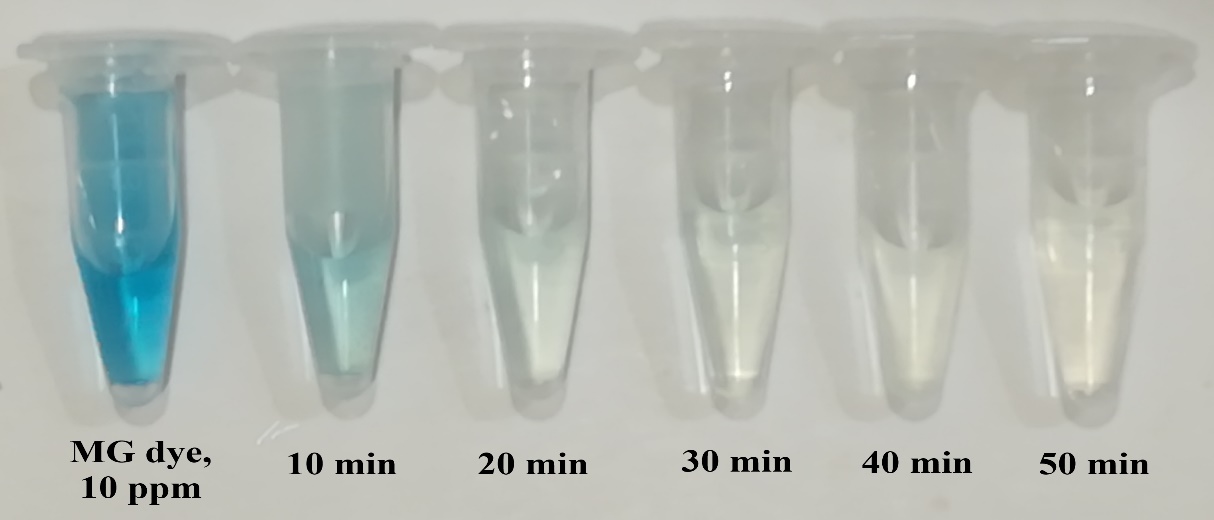


Fig. S6. Representation of the gradual degradation of MB, CV, and MG dye (color change) in the presence of biosynthesized Au@Fe_3_O_4_ BNPs.





Fig. S7. Absorption spectra of adsorption of (a) MB, (b) CV, and (c) MG dye treated with Fe_3_O_4_ NPs.





Fig. S8. Absorption spectra of adsorption of (a) MB, (b) CV, and (c) MG dye treated with Au@Fe_3_O_4_ NPs.

Table S7. Photodegradation and adsorption of MB, CV, and MG dye using Fe_3_O_4_ NPs and Au@Fe_3_O_4_ BNPs.

| Method |  | Photocatalytic degradation (%) |  | Adsorption  (%) | Time |
| --- | --- | --- | --- | --- | --- |
| Sample |  | Fe_3_O_4_ NPs | | |  |
| MB dye |  | 95.24 |  | 58.26 | 70 min |
| CV dye |  | 99.37 |  | 86.57 | 50 min |
| MG dye |  | 96.23 |  | 68.90 | 60 min |
| Sample |  | Au@Fe_3_O_4_ BNPs | | |  |
| MB dye |  | 97.09 |  | 52.26 | 50 min |
| CV dye |  | 99.11 |  | 77.12 | 30 min |
| MG dye |  | 98.04 |  | 46.73 | 50 min |

| Catalyst | Dye | Time | Photocatalytic degradation (%) | Reference |
| --- | --- | --- | --- | --- |
| gold/silver bimetallic nanoparticles | MB dye | 3 h | 34.70 | (Das et al., 2023) |
| Co_0.4_Cd_0.6_Fe_2_O_4_ | MB dye | 90 min | 97.48 | (Vishnu et al., 2023) |
| Sr doped SnO_2_ nanoparticles (1.5%) | CV dye | 150 min | 67.80 | (Kaur et al., 2023) |
| Ni doped SrFe_12_O_19_ nanoparticles | CV dye | 90 min | 91.00 | (Irshad et al., 2022) |
| cobalt oxide nanoparticles | CV dye | 45 min | 64.00 | (Saravan et al., 2020) |
| Ag@Fe bimetallic nanoparticles | MG dye | 180 min | 91.23 | (Sudhakar, Selvam, Poonkothai, & Ranjitha, 2024) |
| Bi@TiO2-rGO nanocomposites | MG dye | 150 min | 93.34 | (Kallawar, Bhanvase, & Sathe, 2023) |
| Zn–Fe co-doped TiO2 | MG dye | 60 min | 91.54 | (Sukhadeve & Gedam, 2023) |
| CuCdS2 nanoparticles | MB dye | 180 min | 68.50 | (Aslam, Saifu Rahman, Shoab, Khan, & Zulfequar, 2022) |
| Ag2O nanoparticles | MB dye | 70 min | 91.5 | (Sagadevan et al., 2023) |
| Fe_3_O_4_ NPs | MB dye | 70 min | 95.24 | Present work |
| Fe_3_O_4_ NPs | CV dye | 50 min | 99.37 | Present work |
| Fe_3_O_4_ NPs | MG dye | 60 min | 96.23 | Present work |
| Au@Fe_3_O_4_ BNPs | MB dye | 50 min | 97.09 | Present work |
| Au@Fe_3_O_4_ BNPs | CV dye | 30 min | 99.11 | Present work |
| Au@Fe_3_O_4_ BNPs | MG dye | 50 min | 98.04 | Present work |

Table S8. The comparison of the photocatalytic activity of Fe_3_O_4_ NPs and Au@Fe_3_O_4_ NPs with some reported materials.



Fig. S9. Reusability test using FTIR spectra of (a) Au@Fe_3_O_4_ NPs (fresh) and reused Au@Fe_3_O_4_ NPs from (b) MB, (c) CV, and (d) MG.

Reference

Aslam, Z., Saifu Rahman, R., Shoab, M., Khan, Z. M. S. H., & Zulfequar, M. (2022). Photocatalytic response of CuCdS2 nanoparticles under solar irradiation against degradation of Methylene Blue dye. *Chemical Physics Letters,* 804, 139883.

Das, G., Seo, S., Yang, I.-J., Nguyen, L. T. H., Shin, H.-S., & Patra, J. K. (2023). Sericin mediated gold/silver bimetallic nanoparticles and exploration of its multi-therapeutic efficiency and photocatalytic degradation potential. *Environmental Research,* 229, 115935.

Irshad, Z., Bibi, I., Ghafoor, A., Majid, F., Kamal, S., Ezzine, S., . . . Iqbal, M. (2022). Ni doped SrFe12O19 nanoparticles synthesized via micro-emulsion route and photocatalytic activity evaluation for the degradation of crystal violet under visible light irradiation. *Results in Physics,* 42, 106006.

Kallawar, G. A., Bhanvase, B. A., & Sathe, B. R. (2023). Sonochemically prepared bismuth doped titanium oxide-reduced graphene oxide (Bi@TiO2-rGO) nanocomposites for effective visible light photocatalytic degradation of malachite green. *Diamond and Related Materials,* 139, 110423.

Kaur, M., Prasher, D., Dhiman, V., Murugesan, P., Ghosh, D., & Sharma, R. (2023). Green energy induced photocatalytic decomposition of methylene blue and crystal violet dyes by strontium doped tin oxide nanoparticles and its antibacterial activity. *Physica B: Condensed Matter,* 661, 414924.

Sagadevan, S., Alshahateet, S. F., Anita Lett, J., Fatimah, I., Poonchi Sivasankaran, R., Kassegn Sibhatu, A., . . . Soga, T. (2023). Highly efficient photocatalytic degradation of methylene blue dye over Ag2O nanoparticles under solar light irradiation. *Inorganic Chemistry Communications,* 148, 110288.

Saravan, R. S., Muthukumaran, M., Mubashera, S. M., Abinaya, M., Prasath, P. V., Parthiban, R., . . . Sagadevan, S. (2020). Evaluation of the photocatalytic efficiency of cobalt oxide nanoparticles towards the degradation of crystal violet and methylene violet dyes. *Optik,* 207, 164428.

Sudhakar, C., Selvam, K., Poonkothai, M., & Ranjitha, S. (2024). Biomimetic synthesis of Ag@Fe bimetallic nanoparticles from Palmyra sprouts extract and their antibacterial, photocatalytic degradation of malachite green. *Inorganic Chemistry Communications,* 161, 112132.

Sukhadeve, G. K., & Gedam, R. S. (2023). Visible light assisted photocatalytic degradation of mixture of reactive ternary dye solution by Zn–Fe co-doped TiO2 nanoparticles. *Chemosphere,* 341, 139990.

Vishnu, G., Singh, S., Kumar Naik, T. S. S., Viswanath, R., Ramamurthy, P. C., Bhadrecha, P., . . . Zahmatkesh, S. (2023). Photodegradation of methylene blue dye using light driven photocatalyst-green cobalt doped cadmium ferrite nanoparticles as antibacterial agents. *Journal of Cleaner Production,* 404, 136977.
